# Post-integration based point-line feature visual SLAM in low-texture environments

**DOI:** 10.1038/s41598-025-97250-6

**Published:** 2025-04-26

**Authors:** Yanli Liu, Zhengyuan Feng, Heng Zhang, Wang Dong

**Affiliations:** School of Electronic Information, Shanghai Dianji University, Shanghai, 201306 China

**Keywords:** Low-texture features, Point-line fusion SLAM, Loop closure detection, Feature extraction, Mathematics and computing, Computer science

## Abstract

To address the issues of weak robustness and low accuracy of traditional SLAM data processing algorithms in weak texture environments such as low light and low contrast, this paper first studies and improves the data feature extraction method, optimizing the AGAST-based feature extraction algorithm to adaptively adjust the extraction threshold according to the gradient size of different data features. Meanwhile, a fusion-based incremental loop closure detection method is proposed, which integrates the similarity scores of multi-dimensional data features based on the Borda counting strategy, thereby enhancing the accuracy of loop closure detection. The performance of loop closure detection was evaluated on public datasets (such as KITTI sequences 00, 05, and 06), achieving an average AP value of 92.03%. The overall system performance was evaluated on the EuRoC dataset, with the results showing a root mean square error range from 0.0061 to 0.0281 m, demonstrating the excellent accuracy and robustness of the proposed method in large-scale data processing.

## Introduction

As data-driven technologies continue to advance, Simultaneous Localization and Mapping (SLAM) has become a cornerstone for understanding and interacting with the surrounding environment, gaining significant attention from both industry and academia^1^. In SLAM, systems typically utilize cameras, LiDAR, or other multimodal sensors to collect environmental data, tracking changes in information over time and space through data association and feature matching.

Feature-based big data processing algorithms are widely adopted for their efficiency and robustness^2^, making them a preferred choice in many applications. Among these, point feature methods are particularly valued for their stability and broad applicability. However, in scenarios with significant lighting variations or low-texture data, point feature descriptions can vary considerably, often resulting in data association failures^3^. Additionally, since most point-based algorithms operate on sparse data, they face challenges in accurately capturing environmental structures in mapping results.In comparison, line features are more robust in structured, artificial environments, being both easier to extract and less affected by environmental conditions^4^. Line features provide a more comprehensive description of structural information, enabling the creation of higher-level environmental maps while simultaneously reducing the number of redundant features, lowering computational complexity, and improving processing speed. However, directly incorporating line feature matching errors into the system can compromise the overall accuracy of data processing.

As a result, big data modeling algorithms that integrate point and line features are gaining increasing attention for their ability to balance robustness and precision^5,6^.

The main contributions of this paper are as follows:

1. The existing point feature extraction algorithm has been improved through an adaptive threshold method, enhancing its robustness in low-texture conditions.
2. A post-integration point-line incremental loop closure detection algorithm is employed to improve the accuracy of loop closure detection in weak texture environments, providing an effective solution for the application of point-line features in SLAM systems.
3. Based on the OBIndex2 incremental bag-of-words model, efficient loop-closure detection with low memory consumption is achieved through binary descriptors and a hierarchical tree structure, meeting the real-time requirements of embedded devices.

## Vision SLAM based on point-line fusion

### Related work

Vision SLAM, widely applied across various fields, leverages visual sensors to gather information about the environment, enabling localization in unknown settings and the construction of environmental maps^7^. Point feature methods have been the dominant choice in the front end of SLAM due to their stability and resilience to changes in lighting and dynamic objects^8^. Mono-SLAM^9^, developed by Davison, is a foundational real-time visual SLAM technology. The system is the first SLAM system based on Extended Kalman Filter (EKF) and Shi-Tomasi features, demonstrating that real-time mapping and localization can be achieved using only a single movable camera as the data source. However, Mono-SLAM struggles with real-time performance in large environments due to the increasing size of the state vector as more feature points are added. To address this issue, Parallel Tracking and Mapping^10^, was designed to reduce the computational overhead of single-camera SLAM systems. ORB-SLAM^11^, which utilizes the ORB keypoint feature descriptor algorithm, and its successor, ORB-SLAM2^12^, known for its robustness and support for stereo and RGB-D cameras, remain among the most widely used methods. In 2021, OV2SLAM^13^, developed by Maxime Ferrera and colleagues, introduced a comprehensive, fully online visual SLAM system capable of real-time operation that balances reduced computational load with exceptional precision and real-time capabilities. ORB-SLAM3^14^, further extending the capabilities of its predecessors, supports fusion with multiple sensors and is the first system to achieve purely visual or visual-inertial fusion.

In natural environments, landmarks such as buildings exhibit distinct structural features and line segments, which are valuable for positioning systems and map construction. Line features offer more advanced information than point features and exhibit superior robustness in feature tracking^15,16^.

Bartoli et al.^17^ laid the groundwork for using line features in SLAM, proposing a way to use line features in motion construction, requiring parameterization methods due to the higher degrees of freedom of line features. They employed Plücker coordinates and standard orthogonal representation, introducing line feature reprojection error. Zhang^18^ built PL-GM using 3D point and line data produced by RGB-D cameras for more accurate indoor dense reconstruction in RGBD-SLAM. Lim et al. addressed structured environment reconstruction using line features and vanishing points but faced limitations in fragmented line features and predominance in indoor scenes. To expedite line feature matching, Wei et al.^19^ proposed a method based on geometric distance, significantly speeding up the process. Ma employed vanishing points (VP) to impose constraints on line features, significantly reducing mismatches^20^. Shao utilized coplanar constraints on line features to eliminate mismatches but lacked real-time data processing^21^. Zuo et al.^22^ developed a powerful SLAM system using heterogeneous point and line features, building an error model with minimal parameters using orthogonal laws. PL-SLAM^23^ used points and line segments throughout many processes, including an extended BoW method considering descriptors of points and lines to improve the loop-closure process. The PL-VINS^24^ system integrates point features, line features, and IMU information to achieve high-precision pose estimation. Simultaneously, it constructs a more intuitive map in the backend using both point and line features. By fusing multi-view visual pose information with IMU navigation data, a redundant visual reprojection error is established. The literature proposes a real-time monocular visual-inertial SLAM with point-line fusion and parallel line fusion. Parallel line groups are extracted by detecting vanishing points in multiple views, and the Point-Line Coupling (PLC) feature, along with tightly coupled PLC residuals of points and lines, enhances the system’s accuracy and robustness. However, there is still room for improvement in terms of runtime efficiency and precision. For target detection and localization in dynamic scenes, TS-BEV (Wang et al., 2024)^25^ proposes a spatiotemporal feature fusion algorithm based on Bird’s Eye View (BEV). This method enhances the robustness of dynamic target detection (e.g., vehicles, pedestrians) by fusing temporal information from multi-frame point clouds and image data, dynamically weighting features at different time steps using an attention mechanism. Compared to traditional single-frame BEV methods, TS-BEV reduces position estimation errors by approximately 18% in complex traffic scenarios, providing significant reference for SLAM systems in dynamic environments. However, its computational overhead is substantial, and it does not fully incorporate geometric constraints from structured features (e.g., line features), limiting its application in low-texture scenes. This paper draws on its spatiotemporal fusion idea within a point-line fusion framework but achieves more efficient environmental modeling through lightweight design and optimization of structured features.

Based on the analysis above, this paper improves the fast AGAST point feature extraction algorithm using an adaptive threshold algorithm for enhanced robustness. It integrates point-line incremental loop closure detection to improve detection accuracy, ensuring stable recognition performance in low-texture feature environments.

### Improved feature point extraction algorithm

**Fig. 1:**
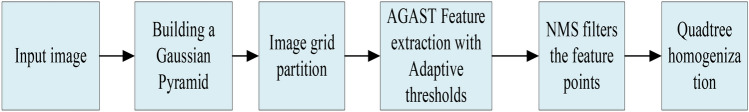
AGAST point feature extraction algorithm with adaptive threshold.

The FAST (Features from Accelerated Segment Test) feature detection algorithm is among the most efficient methods for extracting feature points in visual SLAM systems^26^. Building on FAST, the AGAST (Adaptive and Generic Accelerated Segment Test) algorithm enhances its performance, providing greater speed and improved results in complex scene images. To enhance the reliability of feature point extraction in environments with low texture, this paper utilizes the AGAST algorithm with an adaptive threshold as shown in Fig. 1.

**Fig. 2:**
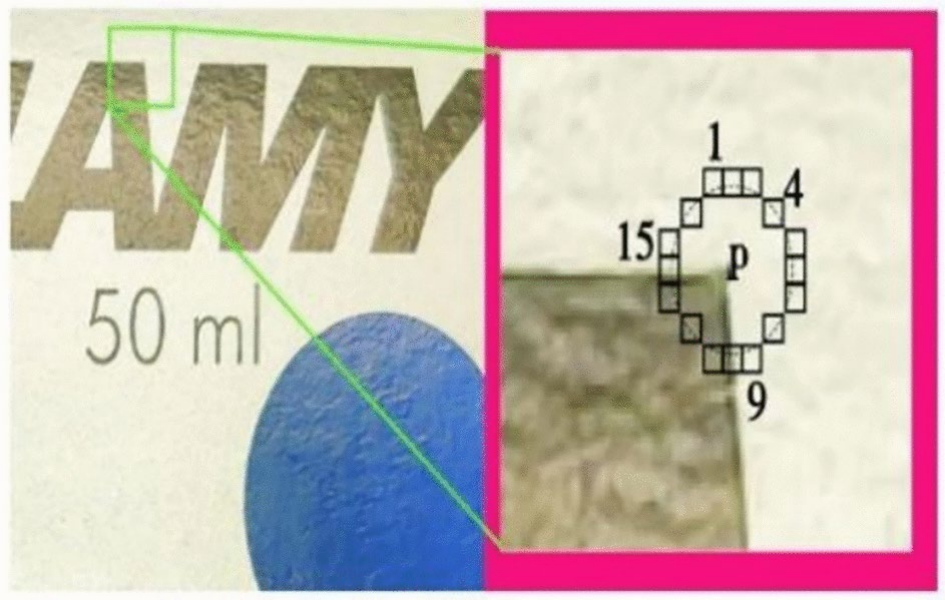
Principle of FAST corner scanning.

**Fig. 3:**
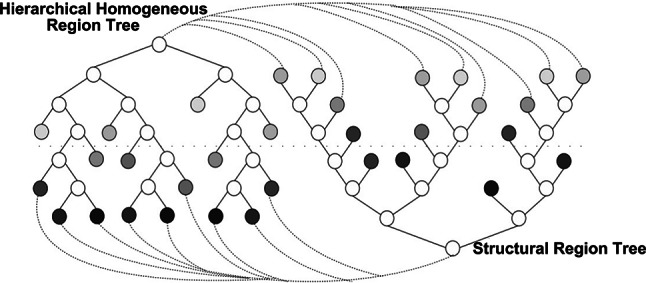
Schematic diagram of AGAST binary tree segmentation principle.

The AGAST algorithm^27^ builds upon the traditional FAST algorithm as shown in Fig. 2, retaining its foundation in the accelerated segment test (AST). It improves the FAST algorithm by replacing the ID3 decision tree with a binary tree, as illustrated in Fig. 3. This modification addresses the slower operation of the ternary tree structure used in the ID3 decision tree. Additionally, when encountering an entirely new environment, the ID3 decision tree requires the reacquisition of environmental data, resulting in reduced detection efficiency.

AGAST offers a more refined matching space to extract sufficient pixels. It uses a 3-pixel radius circle centered on the detection point as a template, identifying pixels with values above or below the neighborhood threshold as feature points. To enhance the localization of these feature points, the algorithm introduces two new configuration spaces, “brighter” and "darker," along with updated allocation rules:

1 $$S_{p \to x} = \left\{ {\begin{array}{*{20}l} {d,I_{p \to x} < I_{p} - t} \hfill \\ {\overline{d},I_{p \to x} \ge I_{p} - t \cap S_{p \to x} = u} \hfill \\ {s,I_{p \to x} \ge I_{p} - t \cap S{\prime}_{p \to x} = \overline{b}} \hfill \\ {s,I_{p \to x} \le I_{p} - t \cap S{}{\prime}_{p \to x} = \overline{d}} \hfill \\ {b,I_{p \to x} > I_{p} - t} \hfill \\ {\overline{b},I_{p \to x} \le I_{p} - t \cap S{\prime}_{p \to x} = u} \hfill \\ \end{array} } \right.$$

In Eq. 1,$I$ represents the brightness of the pixel,$S^{\prime}_{p \to x}$ is the previous state,$d$ denotes pixels darker than the central pixel,$\overline{d}$ are pixels brighter than the central pixel,$s$ indicates similarity to the central pixel,$b$ are bright points,$\overline{b}$ are points darker than the central pixel,$u$ represents an undefined state, and $t$ is the threshold.

**Fig. 4:**
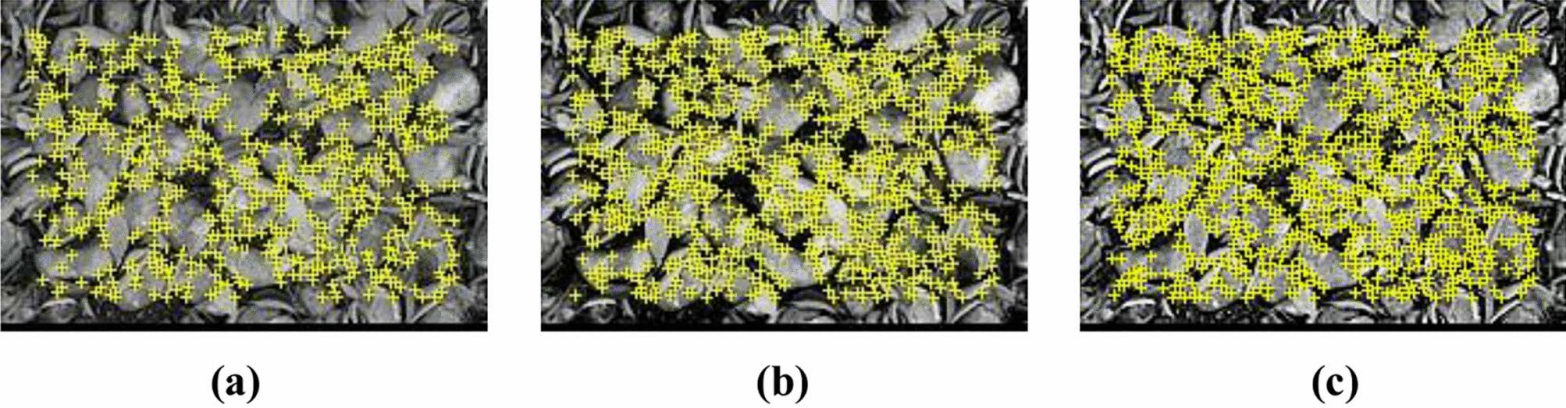
Distribution of corners without the use of adaptive thresholding.

The threshold quality directly impacts the selection of feature points. A larger threshold results in fewer selected feature points, while a smaller threshold increases the number of selected points. The traditional AGAST corner extraction algorithm employs a constant threshold, which, though convenient, can lead to errors in feature point extraction when lighting or contrast conditions vary. As illustrated in Fig. 4, under intense lighting or high contrast, more corners are detected, whereas dim lighting or low contrast reduces the number of detected corners, thereby affecting the decision tree training.

2 $$T = \alpha \frac{{\left[ {\sum\limits_{n = 1}^{16} {I_{i} - I_{MAX} - I_{MIN} } } \right]}}{{I_{AVER} }}$$

Therefore, this paper designs a method with an adaptive threshold, which shows a certain degree of adaptability under different lighting and contrast conditions. In Eq. 2, Let the threshold be $T$, where $\alpha$ represents the adaptive factor, $I_{MAX}$ and $I_{MIN}$ are the maximum and minimum values of the 16 pixels sorted by grayscale value, and $I_{AVER}$ is the average grayscale value of the pixels on the circle, excluding the maximum and minimum pixels. This paper finds that in low-contrast regions (such as white walls), the range $IMAX - IMIN$ is small, and the threshold is automatically reduced to $T \approx 8 - 12$ to avoid missed detections; in high-contrast regions, the threshold is increased to $T \approx 25 - 30$ to suppress noise points. To improve the detectability of low-texture images, CLAHE with a block size of 8 × 8 and a clip limit of 2.0 is applied to the input images to enhance local textures. After smoothing with a Gaussian kernel of size $\sigma = 1.0$, Laplacian edge enhancement is superimposed, and the formula 3 is:

3 $$Ienhanced = IGaussian + 0.5 \cdot Laplacian(IGaussian)$$

**Fig. 5:**
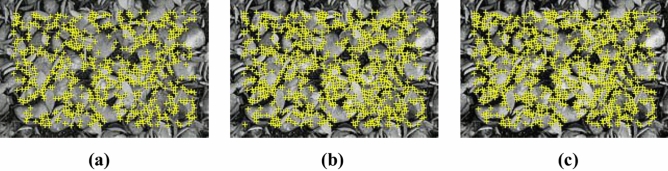
Corner distribution with adaptive threshold.

As shown in Fig. 5, it is evident that after the adjustment of the adaptive threshold, the number of corner points increases in images with low contrast and decreases in images with high contrast. Overall, the distribution of corner points becomes more stable, which is conducive to the overall training of the decision tree.

### Visual odometry with integrated point and line features

**Fig. 6:**
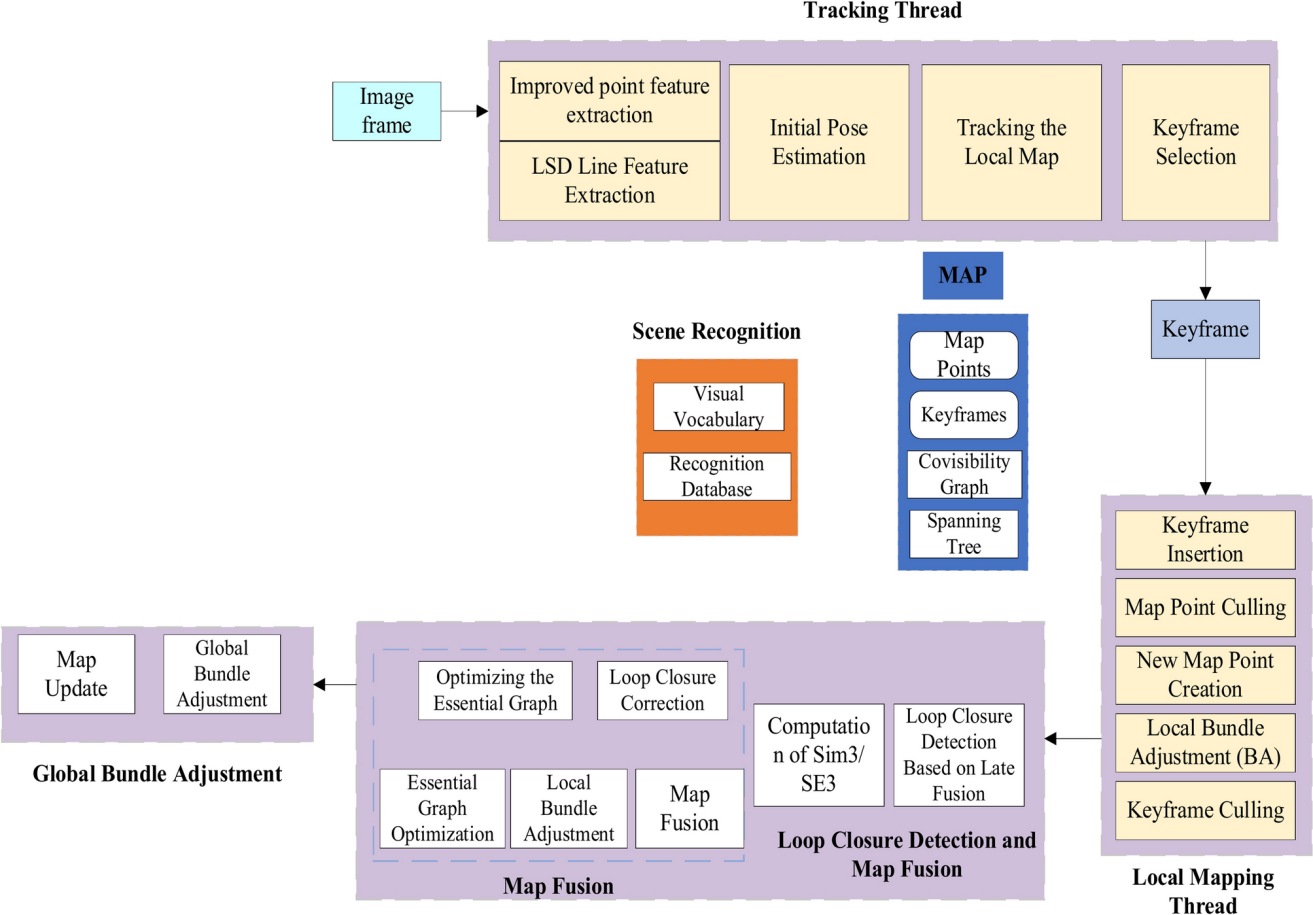
Visual SLAM framework integrating point and line features.

To overcome the challenge of insufficient point feature extraction in indoor low-texture scenes, this paper introduces a post-integration point-and-line incremental loop closure detection method derived from the adaptive threshold AGAST algorithm. This method enhances the precision of loop closure detection in environments with low texture, such as those with dim lighting. The overall system operation structure is shown in Fig. 6.

**Fig. 7:**
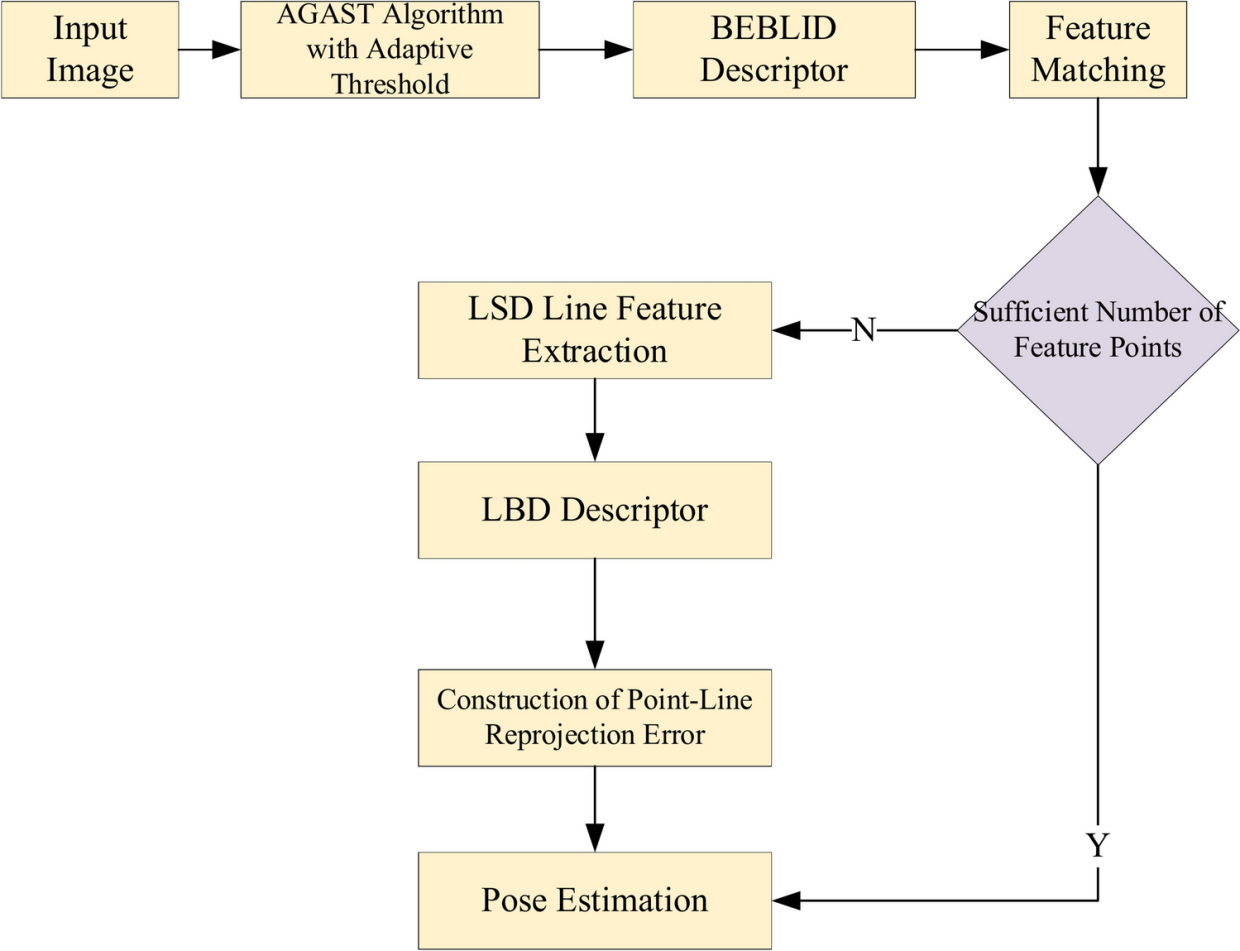
Flowchart of visual odometry.

Given the stable performance of LSD line features in low-texture conditions, this paper integrates the enhanced point feature matching algorithm with line features. By integrating the enhanced point feature extraction algorithm with line features, a visual odometry system is proposed that merges both point and line features. Figure 7 illustrates the process flow of the improved visual odometry.

#### Line feature extraction and matching

**Fig. 8:**
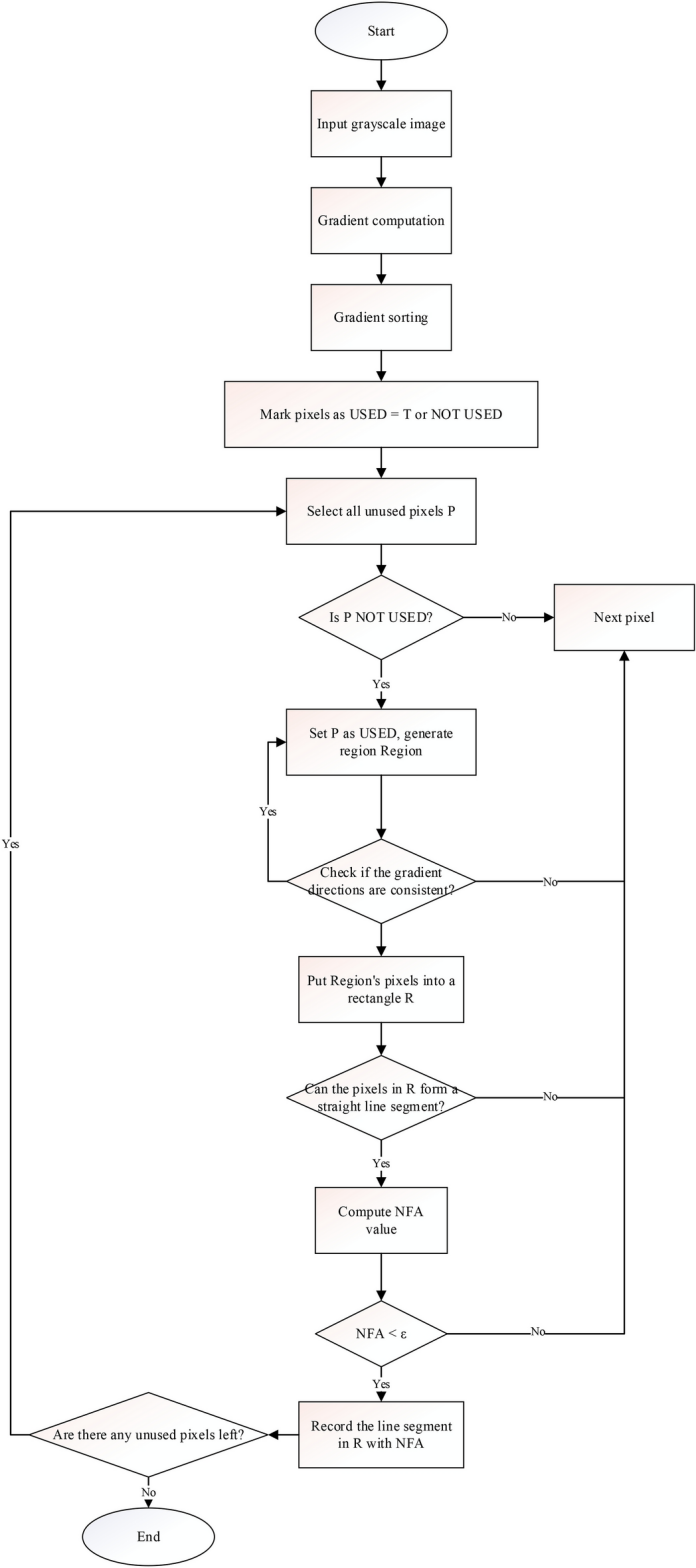
LSD line segment detection process flow diagram.

Line segments, as geometric primitives, have a dual relationship with points and can be effectively utilized in SLAM systems, much like points. Standard line feature extraction algorithms include Canny^28^, Hough Transform^29^, and EDlines^30^. In SLAM systems, obtaining geographic information from the environment is essential, and straight lines are among the key features commonly present in many environments. The introduction of the LSD (Line Segment Detector) algorithm allows robots to more accurately perceive and understand the line structures in their surroundings, thereby improving the efficiency and reliability of SLAM systems. The LSD^31^ line detection algorithm is designed to extract high-quality line segment features from sensor data, such as those provided by cameras or LiDAR. Unlike traditional edge detection algorithms, LSD is specifically optimized for detecting and parameterizing straight lines in the environment. Figure 8 displays the detailed workflow of the LSD algorithm.

The LSD line detection algorithm is based on the brightness and gradient information of the image to find line segments. The main parts of the algorithm are:

1. Use Gaussian smoothing to remove noise.
2. Compute gradients for the input image, calculating gradient values and directions of pixels.
3. Build rectangles in the direction of the principal inertia axis of the support domain, using the gradient magnitude of the pixels as the quality measure to determine the centroid and direction of the rectangle, which contains all points in the region.
4. Calculate the density of points within the rectangular Area, assessing whether it meets the set threshold. If not, truncate the Area and finally determine whether it conforms to the conditions of a straight line.

The original LSD algorithm fixes the gradient threshold at $\tau = 8$, whereas this paper dynamically adjusts it based on the image’s average gradient $\overline{G}$ as shown in formula 4:

4 $$\tau = \max (5,0.5 \cdot \overline{G} )$$

In low-texture images($\overline{G}$ < 10), the threshold is reduced to 5 to retain more line segments. Regarding line segments, this paper enforces the retention of segments with a length ≥ 15 pixels to avoid the interference of fragmented short segments in matching. If a dominant direction exists in the scene (e.g., parallel lines in a corridor), vanishing points are fitted using RANSAC, and only line segments with a directional deviation < 5° are retained.

**Fig. 9:**
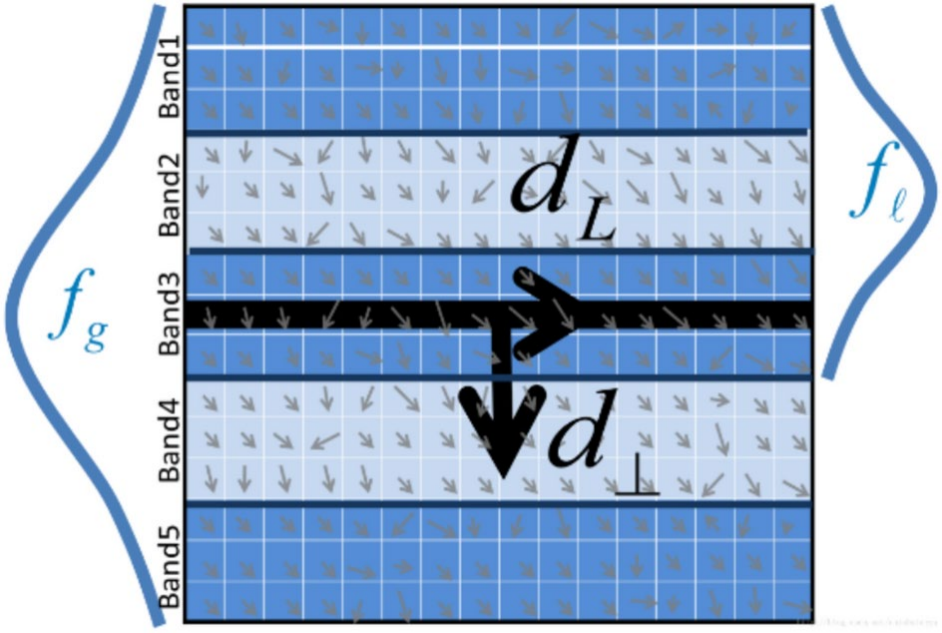
LSR support domain partition diagram.

LBD descriptors are used for line feature matching. The core idea of LBD is to divide the local Area into several sub-regions or cells. For each sub-region, the histogram of gradient orientations is calculated. This histogram will contain information about the distribution of gradient orientations. The information from the gradient histogram is encoded into a binary representation. Specific values in the gradient histogram are compared with a specific threshold, and the results are converted into binary bits^5^. The specific process of the LBD descriptor is as follows: First, construct the scale space and extract line segments, which constitute the LSR (Line Support Region) line segment support domain, as shown in Fig. 9:

$\{ B_{1} ,B_{2} ,...,B_{n} \}$ is a subregion of the LSR support domain, obtained by dividing the LSR support domain. $d_{L}$ represents the direction of the line, and $d_{ \bot }$ is perpendicular to the direction of the line in a clockwise direction. Both global and local Gaussian functions are applied in the $d_{ \bot }$ direction for each row.

In Eq. 5, By concatenating each band descriptor $BD_{j}$ using the LBD descriptor,

5 $$LBD = (BD_{1}^{T} ,BD_{2}^{T} ,...,BD_{n}^{T} )$$

can be obtained.

$BD_{j}$ can be derived from two adjacent bands, $B_{j}$ and $B_{j - 1}$, in $B_{j + 1}$. This paper addresses low-texture scenes by performing binarization compression, using the mean of the gradient histogram of each sub-region as the binarization threshold. If the histogram kurtosis is < 3.0 (indicating a flat distribution), the sub-region is ignored to reduce noise interference.

#### Point and line reprojection error and backend optimization

**Fig. 10:**
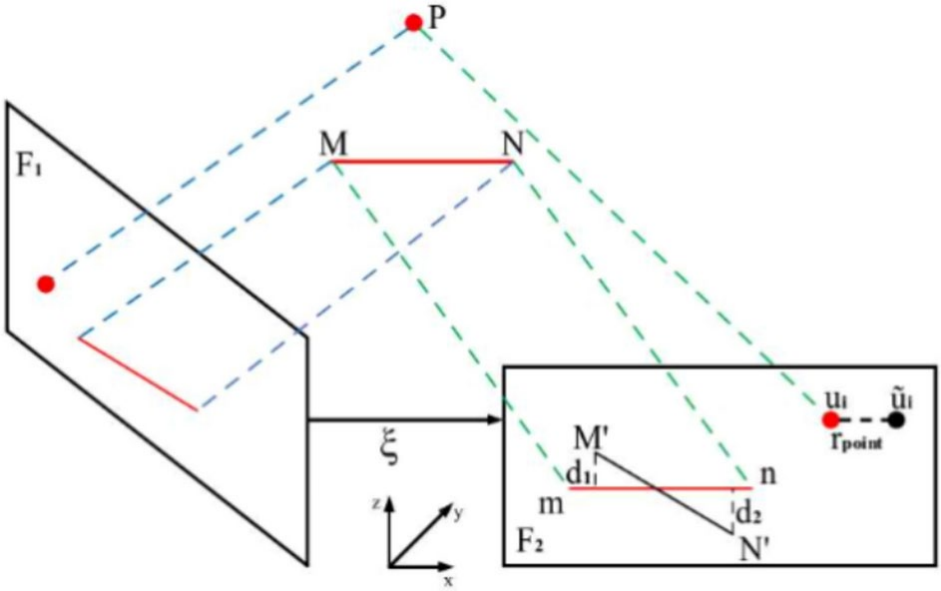
Point and line reprojection error diagram.

After completing point and line feature matching, it is necessary to construct and optimize a reprojection error model for these features. The purpose of this step is to ensure that the estimated map and camera pose are consistent with the actual scene. To achieve this goal, the system needs to reproject the extracted feature points and line features back onto the image plane and compare them with the actually observed features. The objective of the local Bundle Adjustment (BA)^32^ optimization is to maximize the consistency and accuracy of the system by adjusting poses and maps. The reprojection error of point and line features is shown in Fig. 10:

The keyframe selection strategy used in this paper is the same as that in ORB-SLAM3. Graph optimization methods, such as G2O^33^, are used to minimize the error function of visual SLAM. These error functions include errors in camera pose, map point locations, and loop closure. By optimizing these error functions, the system can more accurately estimate the camera trajectory and the location of map points, thus improving localization accuracy. Reprojection error is also used to optimize the camera trajectory and map point positions. This is a pixel-level error measurement that optimizes by minimizing the error between observed feature points and their estimated locations on the map.

### Post-integration point-line incremental loop closure detection

**Fig. 11:**
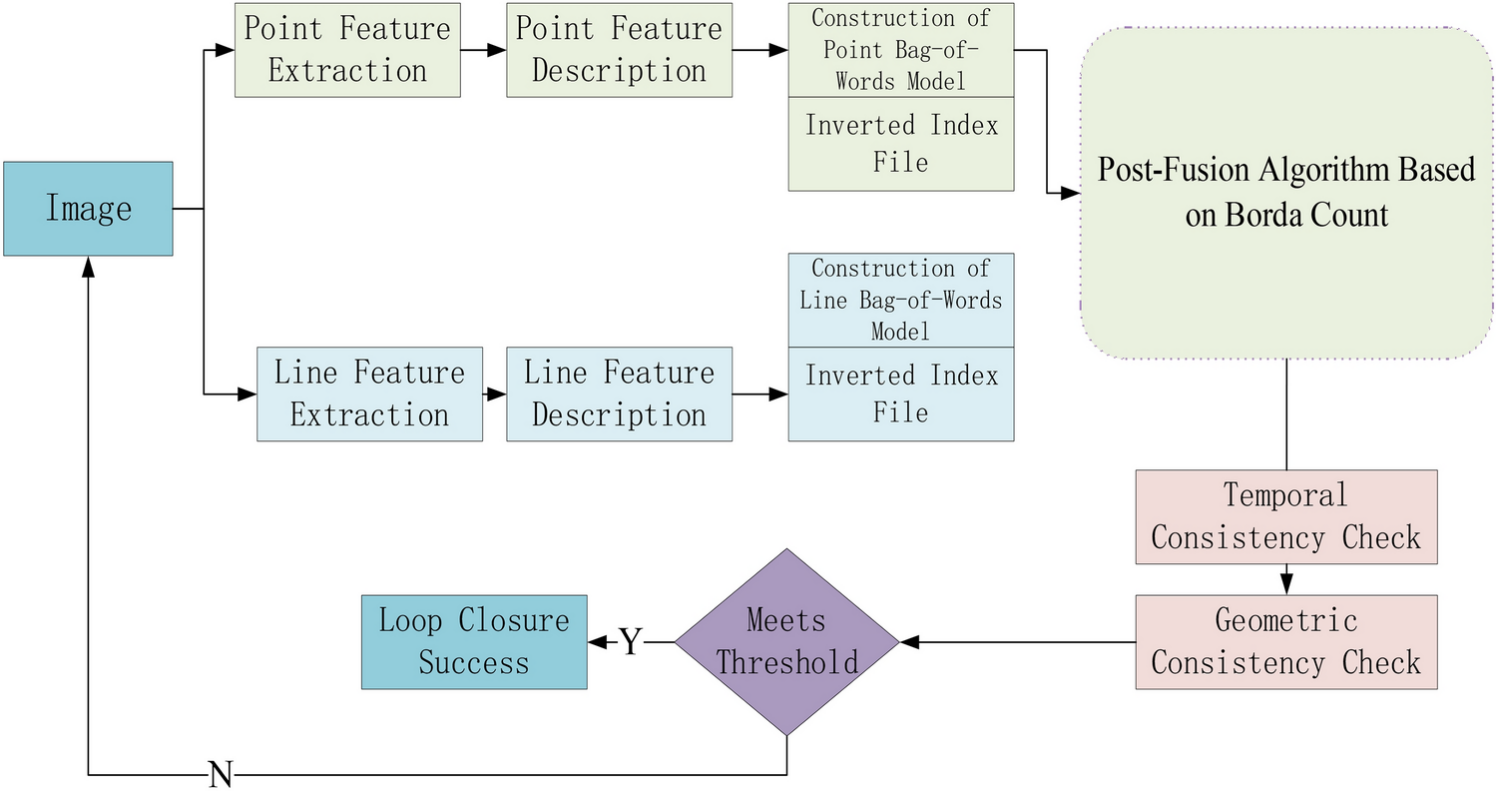
Flowchart of the loop closure detection method.

Conventional loop closure detection often employs point feature descriptors^34^. In low-texture environments, it can be challenging to find sufficient point features, leading to decreased algorithm performance. Against this backdrop, this paper proposes a post-integration point-line incremental loop closure detection method. To reference previous images, the paper utilizes distinct incremental binary bags of words (BoW) for point and line features. A novel post-integration method is used to merge candidate images from each BoW instance. The flowchart of the suggested loop closure detection approach is illustrated in Fig. 11.

In each processed image, two sets of features are identified: point and line features. Since both feature types are described using binary descriptors, similarity calculations are performed separately for each. The extraction and description of point features employ the improved AGAST algorithm, while line features are extracted using the LSD. In the later fusion algorithm, Borda’s point-line similarity scoring method is utilized to determine the final similarity score, achieving the fusion of point and line features. The objective of loop closure detection is to ascertain whether the current location matches a location previously visited by the robot. To facilitate loop closure detection, a database of images is continuously compiled as the robot captures new images, storing information from previously acquired images^35^. For every input image, the paper employs a multi-level tree structure to retrieve candidates for loop closure detection efficiently. This structure can manage an increasing number of visual words and enable incremental operations.

#### Construction of the incremental bag-of-words model

In this paper, point feature detection is performed using the adaptive AGAST algorithm, and the features are described using the binary BEBLID descriptor^36^. Line features are obtained using the LSD algorithm, while line segments are described using the binary form of LBD. Both key point and line segment descriptors are used simultaneously, aiming to enhance the accuracy of retrieval results in a broader range of scenes. This is particularly effective in contexts where line segment descriptors may provide a more precise representation of the environment than point descriptors, especially in scenes with weak textures but strong structures. This combination can adapt to different types of environments and image characteristics better, thereby improving the robustness of image retrieval.

To effectively manage the increasing number of visual vocabulary, an efficient indexing scheme is needed. Normally, this problem can be solved by using methods like KD Tree or K-means^37,38^, but these methods are usually not applicable to binary descriptors. This paper adopts OBIndex2^39^ as the incremental bag of words model for processing binary descriptors. Combined with reverse indexing, OBIndex2 can quickly perform image retrieval. The initialization and construction process of the index is as follows: Initially, the index is constructed using the descriptor of the first image as the initial visual vocabulary. This means that the descriptor of the first image is used as the initial visual word. Next, when new images are added to the index, their descriptors must be searched to find their corresponding positions within the index. For a given binary descriptor query, find the two closest neighbors by traversing the tree from the root to the leaf nodes, and selecting the node at each level that minimizes the Hamming distance. Then use a threshold to determine whether these two descriptors represent the same visual features. If so, merge the query descriptor with the visual vocabulary and replace the visual vocabulary with the new descriptor. If not, treat the query descriptor as a new element and incorporate it into the database as a new visual term. In either case, the reverse index will be revised, appending the current image’s index to the list associated with the updated or newly added term. Finally, when the features of the query image are provided as input, OBIndex2 generates a ranked list of images using a scoring system based on TF-IDF weighting^40^. This index construction and retrieval process is used to manage a large number of image descriptors, especially for handling binary descriptors, to achieve efficient image retrieval.

OBIndex2 combines incremental binary bag of words scheme and reversed files to quickly get similar images. OBIndex2 uses a multi-level tree structure to efficiently handle the growing number of visual words. This paper maintains two OBIndex2 instances: one for processing point features and the other for processing line features. For each instance, a progressively built visual vocabulary is created, along with an image index for every feature. When an image is provided, a simultaneous search is conducted on each index to find the image with the closest matching points and lines. Assuming two lists are retrieved, among which the most similar image of points is retrieved: $C_{p}^{t} = \{ I_{{p_{0} }}^{t} ,...,I_{{p_{a - 1} }}^{t} \}$, and the most similar image of line b was retrieved: $C_{l}^{t} = \{ I_{{l_{0} }}^{t} ,...,I_{{l_{b - 1} }}^{t} \}$. Each list is arranged based on their similarity ratings, denoted as $S_{p}^{t} (I_{i} ,I_{j}^{t} )$ and $S_{l}^{t} (I_{i} ,I_{j}^{t} )$, which assess the similarity between keyframes $I_{i}$ and $I_{j}$. Each list is sorted according to their respective correlation scores $S_{p}^{t} (I_{i} ,I_{j}^{t} )$ and $S_{l}^{t} (I_{i} ,I_{j}^{t} )$, which are used to assess the similarity between $I_{i}$ and $I_{j}$. These scores are calculated using the Term Frequency-Inverse Document Frequency (TF-IDF). Subsequently, these scores are normalized to a range of [0, 1], which helps to manage score range variations caused by the distribution of each visual word. The method of calculation is shown in Eq. 4. Finally, to control the number of candidates in each list, images with normalized scores $S$ below a predetermined threshold are filtered out.

6 $$S = \frac{{S_{pl}^{t} (I_{i} ,I_{j}^{t} ) - S_{pl}^{t} (I_{i} ,I_{min}^{t} )}}{{S_{pl}^{t} (I_{i} ,I_{max}^{t} ) - S_{pl}^{t} (I_{i} ,I_{min}^{t} )}}$$

As shown in formula 6, $S_{pl}^{t} (I_{i} ,I_{max}^{t} )$ and $S_{pl}^{t} (I_{i} ,I_{min}^{t} )$ represent the minimum and maximum scores, respectively, for the loop closure candidate image lists. $S_{pl}^{t}$ is the combined score list, merging the scores from $S_{p}^{t}$ and $S_{l}^{t}$.

#### Post-integration algorithm

After obtaining the two lists $C_{p}^{t} = \{ I_{{p_{0} }}^{t} ,...,I_{{p_{a - 1} }}^{t} \}$ and $C_{l}^{t} = \{ I_{{l_{0} }}^{t} ,...,I_{{l_{b - 1} }}^{t} \}$ for point and line features, the next step is to merge them to acquire an overall view of the loop closure candidate frames. However, it is necessary to consider both point-based and line-based loop closure candidate frames simultaneously. Several techniques are available for merging this kind of multimodal data, commonly divided into two categories: early fusion and post-integration. Early fusion refers to the process where all features from different modalities are combined into a single form before similarity calculation. For instance, descriptors of point features and line features can be stacked together. Since both point and line features are represented as 128-bit binary descriptors, they can be fused at the descriptor level to form a single 256-bit binary descriptor. This means that the features of lines and points are combined into a unified image descriptor, which is then used for similarity calculation and decision-making. In contrast, post-integration is processed at the decision level. It involves combining the outputs (similarity scores) of the dual-modal point-line features.

In this paper, a voting algorithm based on Borda count is used to merge the lists of loop closure candidate key frames. The fundamental concept is as follows: Firstly, a group of voters ranks a fixed list of candidates according to their preferences. Next, scores are allocated inversely proportional to the candidates’ rankings. Finally, once all the votes are cast, the candidate with the highest total score wins. In this paper, there are two independent groups of voters, one representing $C_{p}^{t} = \{ I_{{p_{0} }}^{t} ,...,I_{{p_{a - 1} }}^{t} \}$ and the other $C_{l}^{t} = \{ I_{{l_{0} }}^{t} ,...,I_{{l_{b - 1} }}^{t} \}$, each issuing their respective ordered lists of loop closure candidate key frames, $C_{pl}^{t} \{ I_{{p_{0} }}^{t} ,...,I_{{p_{a - 1} }}^{t} ,I_{{l_{0} }}^{t} ,...,I_{{l_{b - 1} }}^{t} \}$. The number of candidate frames to be voted on is $m$, where $m$ is the minimum length of the two lists, denoted as $min(a,b)$. The top $m$ images in each list $C_{pl}^{t} \{ I_{{p_{0} }}^{t} ,...,I_{{p_{a - 1} }}^{t} ,I_{{l_{0} }}^{t} ,...,I_{{l_{b - 1} }}^{t} \}$ are ranked and assigned a score, $S_{pal}$ in Eq. 7.

7 $$S_{pal} (I_{i}^{t} ) = (m - c)S$$

where $i$ signifies the order of image $I_{i}$ in the list, and $S$ is the standardized rating of the image in the list, as calculated in the previous section. When the same image appears in both keyframe lists, the formula 8 is used to calculate its score:

8 $$S_{s} (I_{i}^{t} ) = \sqrt {S_{p} (I_{i}^{t} )S_{l} (I_{i}^{t} )}$$

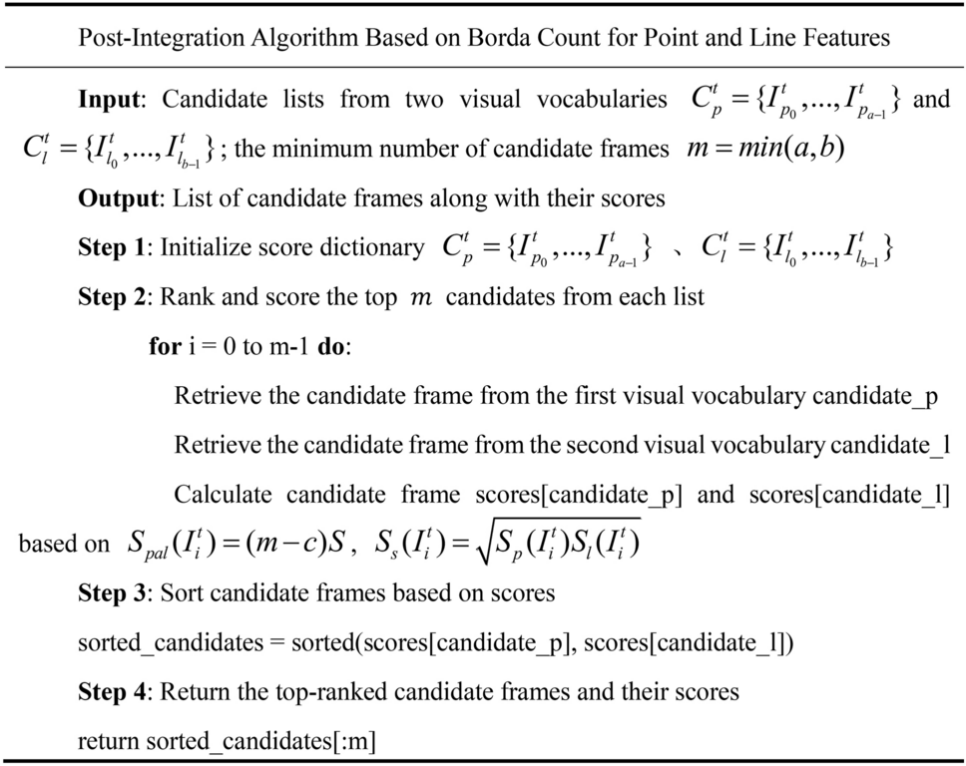


The method uses a geometric way to reduce the impact of incorrect matches in one of the lists. This allows the information in both lists to be sorted according to the score $S_{s} (I_{i}^{t} )$, obtaining a fused candidate frame list that integrates both point and line features. Additionally, there’s a special case for images that only have either point or line features. For these keyframes, as they appear only in one of the ordered lists, they are directly placed into the final fused scoring list for sorting. The above process describes the point-line post-integration algorithm based on the Borda count.

#### Algorithm robustness analysis

In low-texture or illumination-varying scenes, traditional SLAM systems relying on point features often suffer from unstable extraction due to insufficient gradients or sudden brightness changes. The adaptive threshold AGAST algorithm and LSD line feature fusion strategy proposed in this paper enhance system robustness through the following mechanisms:

The traditional AGAST algorithm employs a fixed threshold ($Tfix$), resulting in too few feature points in low-contrast scenarios (Fig. 4a) and excessive redundant points in high-contrast scenarios (Fig. 4c). As shown in formula 9, this paper introduces an adaptive threshold:

9 $$Tadaptive = \alpha \cdot (Imax - Imin) + \beta \cdot \mu filtered$$

Among them, $Imax$ and $Imin$ are the local region extremes, $\mu filtered$ is the grayscale mean after removing the extremes, and $\alpha$ and $\beta$ are the weighting coefficients. This formula dynamically adjusts the threshold to maintain a balanced number of feature points under varying lighting conditions (Fig. 5).

The gradient adaptivity lies in capturing local contrast through the range $Imax - Imin$, lowering the threshold in low-contrast scenarios to retain more feature points. Noise suppression is achieved by the mean term $\mu filtered$, which filters out abnormal brightness interference and prevents the threshold from being overly sensitive to noise. As a result, the stability of feature extraction is improved, providing a reliable data foundation for subsequent matching and optimization.

The robustness of line features in low-texture environments stems from their geometric invariance: the coordinates and orientation of line segment endpoints are determined by geometric structures and are less affected by brightness variations. In weak-texture regions (e.g., white walls, corridors), point features are sparse, but the LSD algorithm can detect long line segments through gradient accumulation (Fig. 8), providing structural constraints. The reprojection error of point features is a pixel-level offset, while the error of line features can be modeled as a weighted combination of endpoint distance and orientation deviation (Fig. 10). The joint optimization of both forms a dual constraint in low-texture scenes, reducing the dependence of pose estimation on a single type of feature.

The late fusion strategy based on Borda scoring (Formula 7) reflects local texture and global structural information by incorporating the similarity scores of point features and line features, respectively. As shown in formula 10, the Borda strategy employs a weighted geometric mean:

10 $$Sfused = \sqrt {Spoint \cdot Sline}$$

It suppresses mismatches from a single modality (e.g., false positives caused by repetitive textures in point features) while retaining cross-modal consistent matches. In low-texture scenes, point features may fail, but line features can still provide valid loop closure candidates through geometric consistency (e.g., parallel line groups, vanishing points), and vice versa. This redundant design significantly enhances the system’s fault tolerance.

The combination of adaptive AGAST and LSD line features forms a synergy across three stages: feature extraction, data association, and backend optimization. The adaptive threshold ensures the density of point features under low-light conditions, while LSD supplements structural information; the binary nature of point-line descriptors (BEBLID/LBD) supports fast Hamming distance calculations, adapting to the incremental bag-of-words model (OBIndex2); the joint minimization of point-line reprojection errors is achieved through the G2O framework, where the Hessian matrix becomes better conditioned due to multi-feature constraints, reducing the risk of the joint optimization falling into local optima.

#### Loop closure posterior verification

##### Temporal consistency check

Temporal consistency checking is a vital step in SLAM for validating the reliability of loop closure detections^41^. Its main goal is to ensure that detected loop closure frames are temporally coherent with previous loop closures, thus preventing the inclusion of false positives. Initially, the most likely loop closure candidates are selected as described in the previous section. For the top candidate loop closure frame, its timestamp or time label is first identified. Then, previous loop closures, usually the ones closest in time, are reviewed. These frames should be temporally close to the candidate loop closure frame. The time difference between the candidate and previous frames is calculated. If the time difference falls within an acceptable threshold, indicating reasonable temporal consistency, the frame passes the temporal consistency check. If the difference exceeds the threshold, suggesting a lack of temporal consistency, the candidate is discarded as an invalid loop closure. Frames that pass this check are confirmed as valid loop closures and can be added to the map, improving consistency and accuracy. This step helps avoid false positives caused by high similarity between adjacent frames, thus ensuring the consistency and precision of the map.

##### Geometric consistency check

The above method does not fully consider the spatial arrangement of features in the image, nor does it address the issue of incorrect loop associations in challenging perceptual aliasing situations, particularly in man-made environments. To overcome this limitation, a geometric consistency check is introduced to ensure the accuracy and reliability of loop associations^42^. A major drawback of the BoW (Bag-of-Words) approach is its disregard for the spatial arrangement of visual words in the image, which can result in incorrect loop associations under conditions of perceptual aliasing. To address this, a geometric consistency check is applied after the temporal consistency check. The purpose of this check is to verify whether the loop associations align with the geometric consistency of camera motion, eliminating incorrect associations. A common method for this check is the RANSAC algorithm, which effectively handles outliers and noise, though its performance may degrade in the presence of excessive outliers or when the camera motion cannot be modeled with a core matrix. In addition to RANSAC^43^, alternative methods, such as Grid-based Motion Statistics^44^ and Locality Preserving Matching, can also perform geometric consistency checks. Unlike GMS, LPM does not rely on various motion models, which could impact performance, especially with a limited number of detected features. For this reason, this paper selects LPM^45^ for geometric consistency checking to guarantee the precision and reliability of loop closure detection. The LPM method demonstrates excellent performance in maintaining the geometric consistency of camera motion and is more effective in dealing with challenges posed by outliers.

## Experiments and analysis

### Dataset and evaluation metrics

In this paper, for the experimental evaluation of the post-integration incremental loop closure detection algorithm, the KITTI dataset^46^ is used. The images in the dataset include urban road scenes under various weather and lighting conditions. Commonly used metrics for evaluating loop closure detection are the Precision-Recall curve and the Area Under the Curve, where P stands for precision and R stands for recall. The Precision-Recall (P-R) curve is generated with precision on the y-axis and recall on the x-axis. The equations 11, 12 used to compute precision and recall are as follows:

11 $$Precision = \frac{TP}{{TP + FP}}$$

12 $$Recall = \frac{TP}{{TP + FN}}$$

For the experimental dataset of the point-line fusion visual SLAM system, the publicly available EuRoC dataset^47^ is used for system testing. The EuRoC dataset is a standard benchmark for visual SLAM research. Provided by ETH Zurich, it aims to support researchers and developers in evaluating the performance of SLAM algorithms. The EuRoC dataset includes multi-sensor data, such as cameras, Inertial Measurement Units (IMUs), and laser sensors. It features a variety of indoor and outdoor scenes, including offices, corridors, and outdoor spaces, to evaluate the effectiveness of SLAM methods in different environments. The EuRoC dataset also offers data from various types of robots and motion platforms, including quadcopters, mobile robots, and vehicles, facilitating research into different types of SLAM applications. Additionally, the dataset includes high-quality sensor data, such as time-synchronized camera images and IMU data, along with real ground truth trajectories and maps, which can serve as a reference point for the performance of SLAM algorithms.

When analyzing the effectiveness of a SLAM algorithm, it’s common to consider multiple aspects, including time consumption, complexity, and accuracy. Relative Pose Error and Absolute Trajectory Error are often used as evaluation metrics.

RPE describes the accuracy of two frame poses over a fixed time difference. It reflects the relative displacement error and relative rotation error of the odometry. The root mean square of RPE can be calculated using Eq. 13.

13 $$RP{E}_{\text{all}}=\sqrt{\frac{1}{N-\Delta t}{\sum }_{i=1}^{N-\Delta t}{\Vert {\mathit{log}}_{e}(({T}_{gi,i}^{-1}*{T}_{gi,i+\Delta }{)}^{-1}({T}_{esti,i}^{-1}*{T}_{esti,i+\Delta }){)}^{V}\Vert }_{2}^{2}}$$

In Eq. 14, if only the translational part is considered:

14 $$RP{E}_{\text{trans}}=\sqrt{\frac{1}{N-\Delta t}{\sum }_{i=1}^{N-\Delta t}{\Vert {\mathit{log}}_{e}(({T}_{gi,i}^{-1}*{T}_{gi,i+\Delta }{)}^{-1}({T}_{esti,i}^{-1}*{T}_{esti,i+\Delta }))\Vert }_{2}^{2}}$$

Among them, $T_{gi,i}^{ - 1}$ and $T_{gi,i + \Delta }^{ - 1}$ represent the true values of the camera pose, while $T_{esti,i}^{ - 1}$ and $T_{esti,i + \Delta }^{ - 1}$ denote the estimated pose values calculated by the algorithm.

The ATE (Absolute Trajectory Error) is the difference between the estimated pose and the true pose, reflecting the accuracy and robustness of the SLAM system. The ATE for the i-th frame is defined in Eq. 15:

15 $$F_{i} : = Q_{i}^{ - 1} SP_{i}$$

In Eq. 16, using RMES to express ATE indicators:

16 $$RME{S}_{ATE}={\left(\frac{1}{m}{{\sum }_{i=1}^{m}\Vert trans({F}_{I})\Vert }^{2}\right)}^\frac{1}{2}$$

### Loop closure performance evaluation

This paper selects sequences 00, 05, and 06 from the KITTI dataset for experimentation. This paper compares two loop closure detection methods, FAB-MAP^48^ and IBOW-LCD^49^, LDSO、PL-SLAM with the proposed point-line incremental loop closure detection algorithm based on post-integration.

**Fig. 12:**
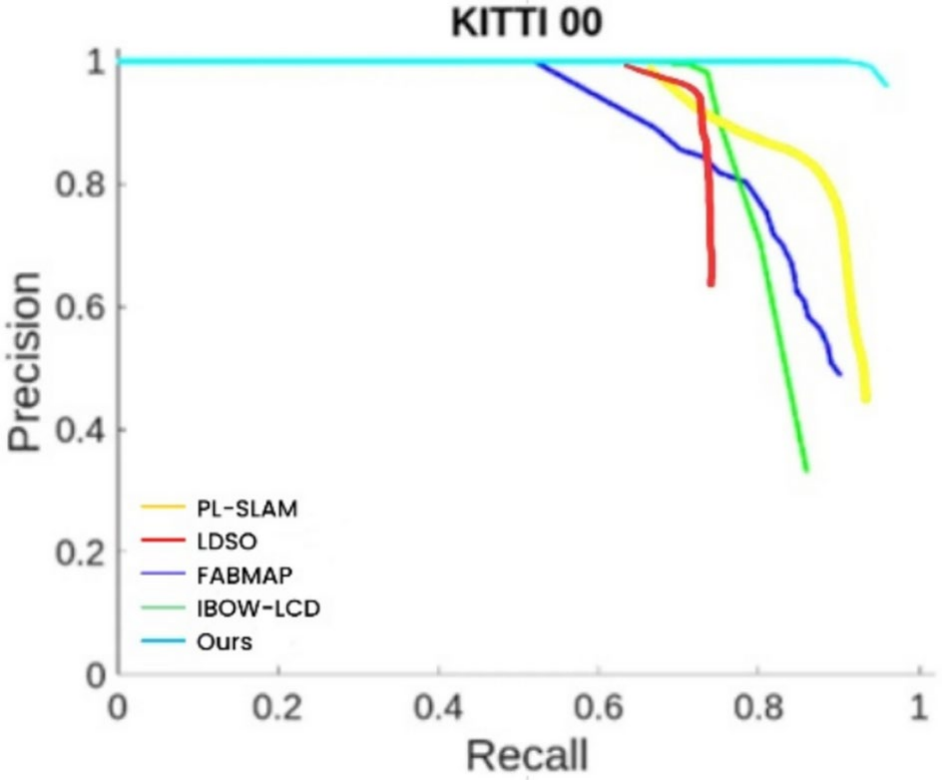
P-R curve for KITTI sequence 00.

**Fig. 13:**
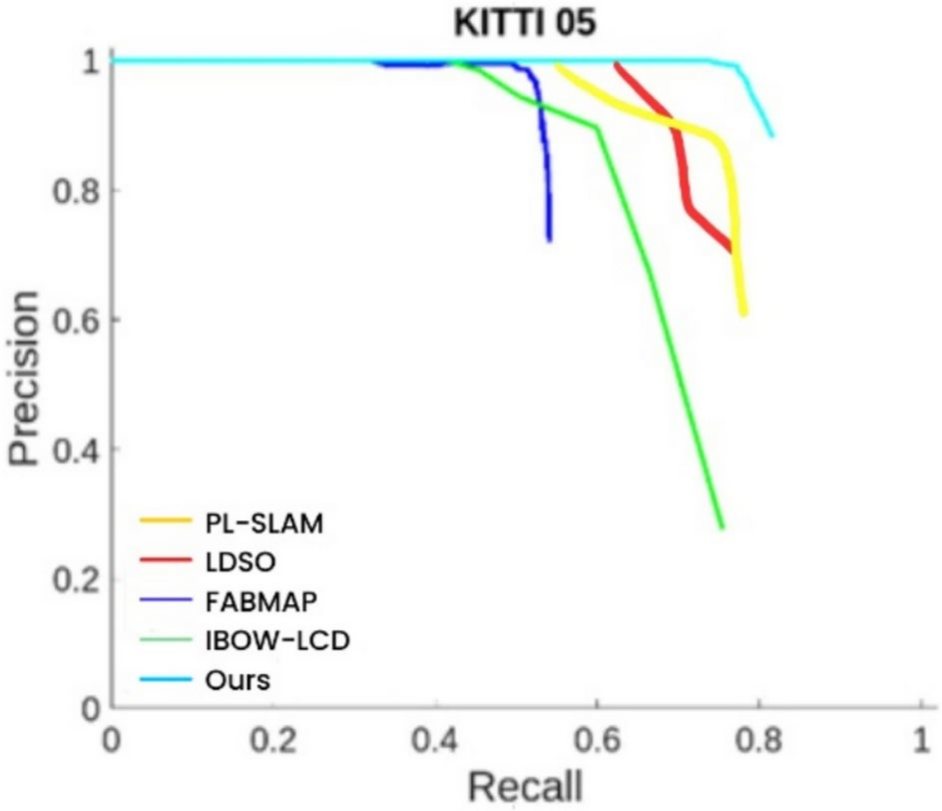
P-R curve for KITTI sequence 05.

**Fig. 14:**
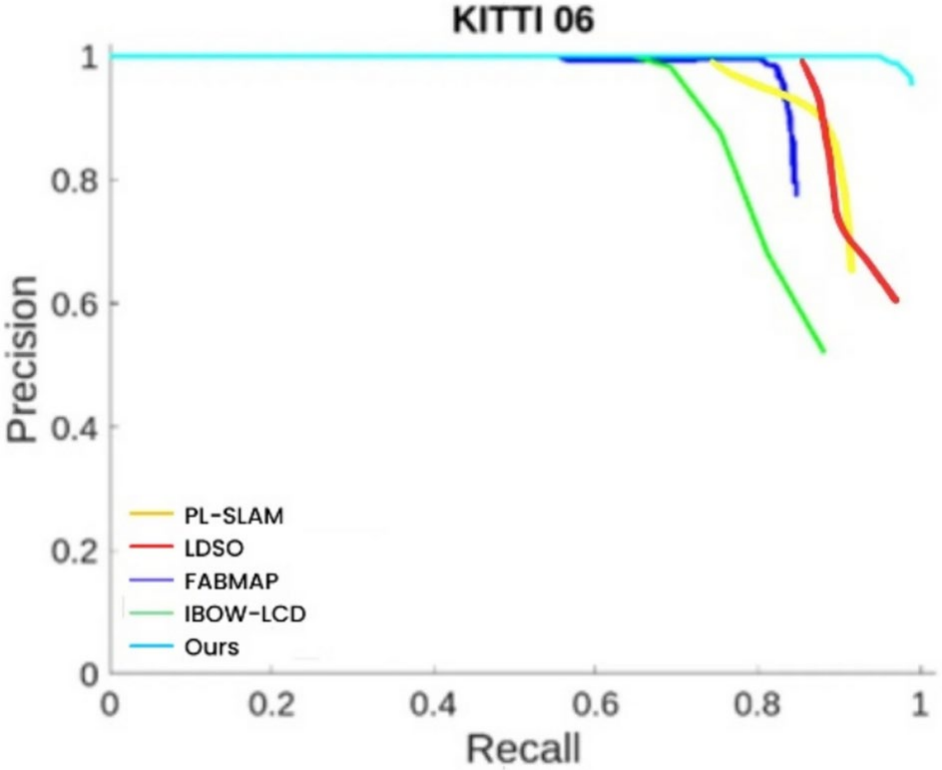
P-R curve for KITTI sequence 06.

From Figs. 12, 13, and 14, it can be observed that the P-R curve of the loop closure detection algorithm proposed in this paper is consistently above the other two algorithms, demonstrating its significant performance advantage on the KITTI dataset. Our algorithm’s P-R curve shows higher performance and better loop closure detection effectiveness compared to the other two algorithms. An analysis and comparison of the performance of these three methods yield the following:

Firstly, from the performance of different algorithms on the P-R curves for sequences 00, 05, and 06, FABMAP achieves high precision at low recall rates. However, as the recall rate increases, its precision drops sharply, indicating that while this algorithm can accurately detect loop closures in certain cases, its overall performance stability is relatively poor. In contrast, IBOW-LCD maintains good precision even at high recall rates, demonstrating its reliability in large-scale recall scenarios. Meanwhile, the P-R curves of PL-SLAM and LDSO reveal their respective strengths. PL-SLAM exhibits relatively stable precision at moderate recall rates, suggesting that it can effectively balance recall and precision in loop closure detection. However, at high recall rates, its precision still falls short compared to IBOW-LCD and the proposed algorithm. On the other hand, LDSO shows a more balanced precision distribution overall, but its performance is slightly inferior to that of PL-SLAM, with lower overall detection accuracy. Most notably, the proposed algorithm maintains high precision across all recall rates in the P-R curve, indicating that it can detect loop closures more accurately in various scenarios, demonstrating superior robustness and global consistency. Its success lies in the adoption of a post-fusion strategy based on points and lines, effectively leveraging the complementary advantages of point and line features, which significantly enhances loop closure detection accuracy. Overall, a comparison of P-R curves clearly shows that the proposed algorithm not only outperforms FABMAP and IBOW-LCD on the KITTI dataset but also exhibits higher precision and robustness than PL-SLAM and LDSO, proving its significant advantages across different scenarios.

While the superiority of our algorithm is evident from the above P-R curves, it is important to note that a detailed quantitative analysis is still required for a concrete evaluation of its performance. Table 1 presents the results of a comparison of the average precision of the three algorithms across sequences 00, 05, and 06.

**Table 1:** Comparison of average precision rates.

Algorithm/Sequence	00 (%)	05 (%)	06 (%)
FABMAP	75.5	53.7	84.4
IBOW-LCD	82.0	68.3	83.6
PL-SLAM	88.3	78.5	89.2
LDSO	79.6	68.3	82.7
OURS	95.9	81.3	98.9

From the data in Table 1, it is evident that the loop closure detection performance of different algorithms varies significantly across different sequences in the KITTI dataset.

Taking Sequence 00 as an example, the proposed algorithm (OURS) achieves the highest AP value of 95.9%, fully demonstrating its exceptional capability in loop closure detection. Among the other algorithms, PL-SLAM achieves 88.3%, IBOW-LCD and FABMAP reach 82.0% and 75.5%, respectively, while LDSO records only 79.6%.For Sequence 05, the proposed algorithm again performs the best, achieving an AP value of 81.3%, significantly surpassing PL-SLAM at 78.5%, IBOW-LCD and LDSO both at 68.3%, and FABMAP at only 53.7%.Finally, in Sequence 06, the proposed algorithm achieves another remarkable result with an AP value of 98.9%. Among the other methods, PL-SLAM attains 89.2%, FABMAP 84.4%, IBOW-LCD 83.6%, and LDSO 82.7%.From the comparison of these AP values, it is clear that the proposed point-line post-fusion incremental loop closure detection algorithm demonstrates superior performance on KITTI sequences 00, 05, and 06, achieving higher loop closure detection accuracy and robustness. It significantly outperforms other commonly used methods, further proving its outstanding performance across different scenarios.

### System performance evaluation combining the improved algorithms from

In this paper, a system for SLAM was designed, and the effectiveness of the enhanced visual SLAM system was assessed. The evaluations were conducted on the EuRoC dataset, using typical sequences MH_01_easy, MH_03_medium, and MH_05_difficult. Comparisons were made against the ORB-SLAM3 algorithm.

**Fig. 15:**
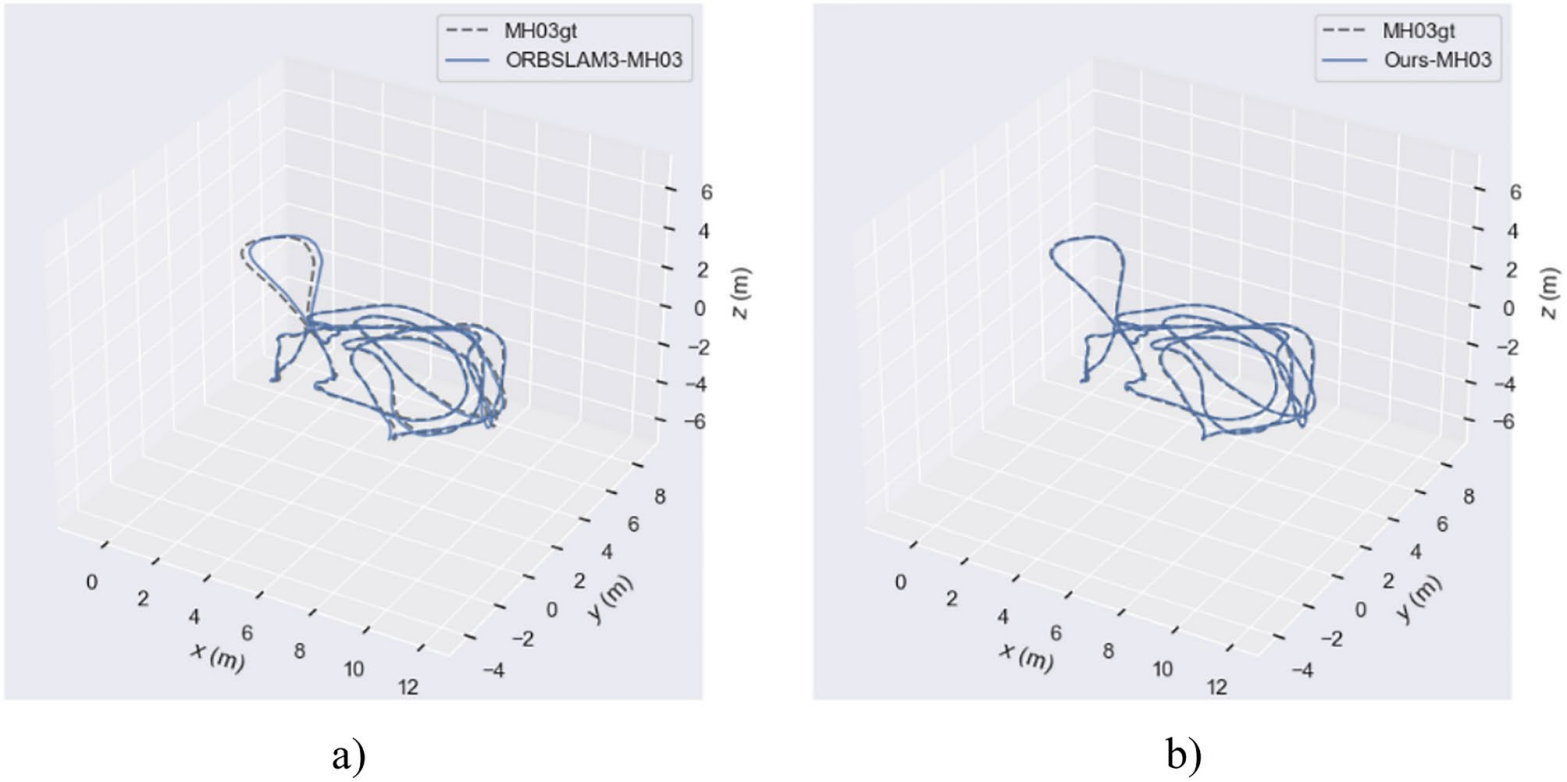
MH_03_medium sequence trajectory.

**Fig. 16:**
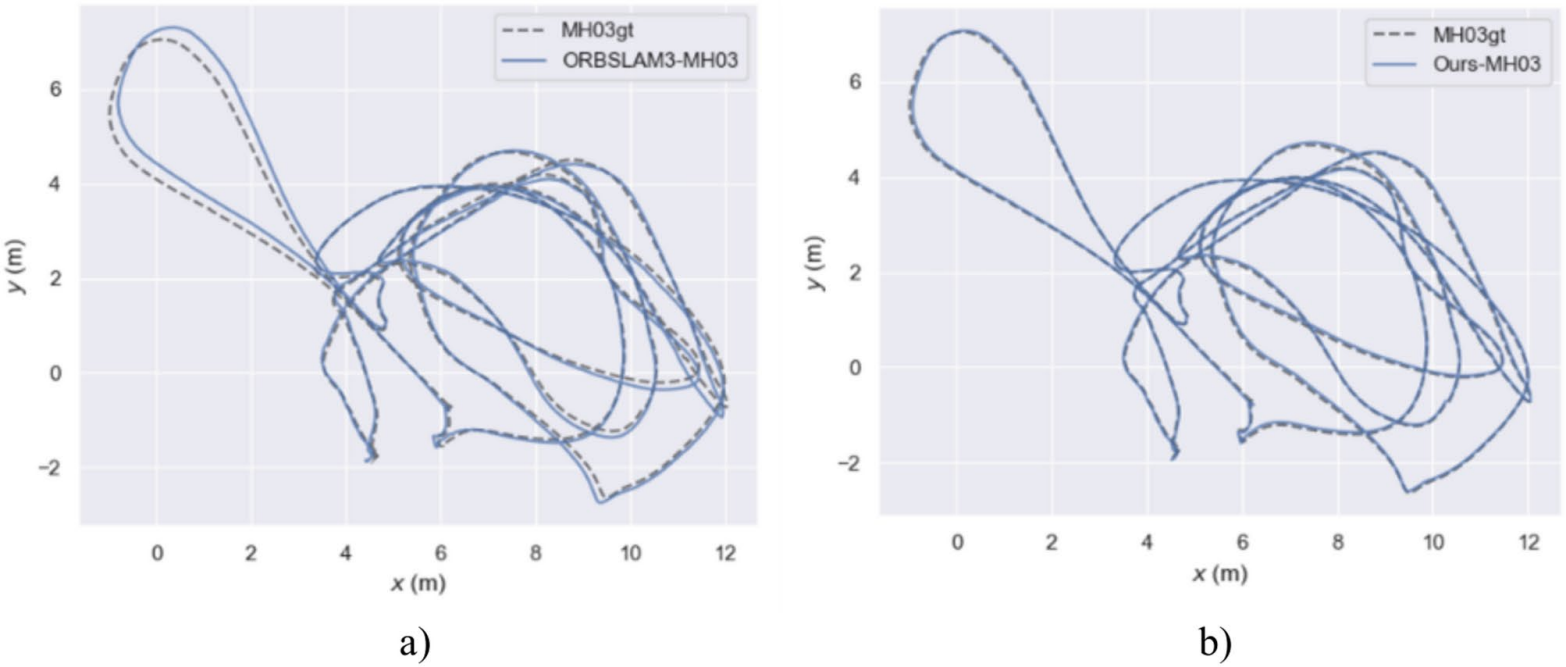
2D trajectory map of the MH_03_medium sequence.

**Fig. 17:**
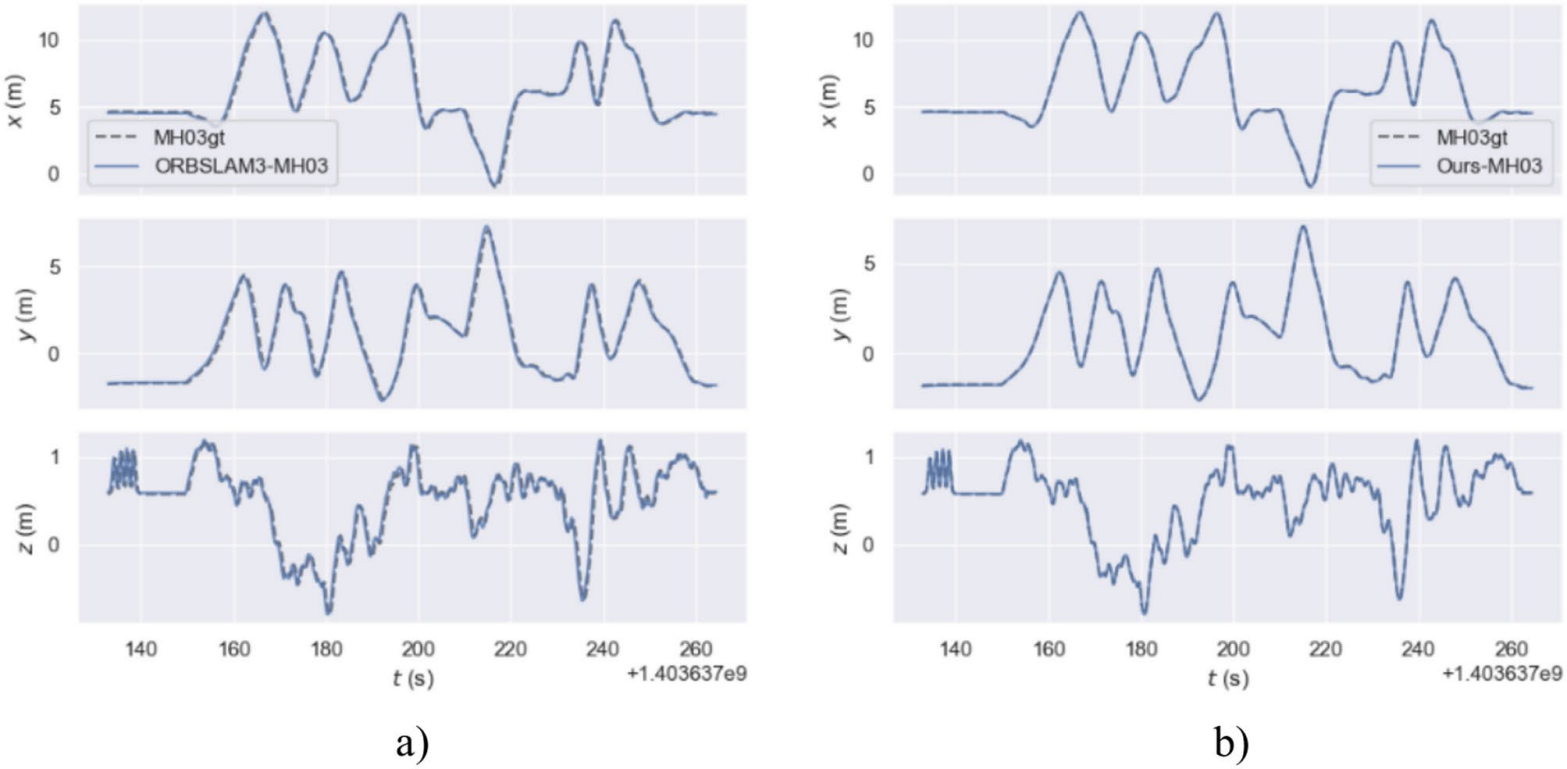
Comparison of three coordinate Offsets in the MH_03_medium sequence trajectories.

Figure 15 describes the true and estimated trajectories of the ORB-SLAM3 algorithm and our method in the MH_03_medium sequence. Figure 16 represents these trajectories on a two-dimensional plane. Through visualizing the trajectories, it is evident that in the MH_03_medium sequence, the trajectory error of ORB-SLAM3 is significantly higher than that of our algorithm. Figure 15b shows that our trajectory is closer to the real path. This indicates our algorithm’s pose estimation is essentially consistent with the real trajectory provided by the dataset, demonstrating overall stability. Additionally, over time, there are slight deviations between the estimated trajectory and the real path. Figure 15b reveals some areas where ORB-SLAM3’s trajectory does not overlap with the real trajectory, indicating larger trajectory errors. Further, Fig. 17 presents a contrast of the three-coordinate deviation results between the two algorithms. From the comparison in Figs. 17a, b, it can be seen that the three-coordinate deviation results of our algorithm are almost consistent with the real values, while those of ORB-SLAM3 show a larger deviation.

**Fig. 18:**
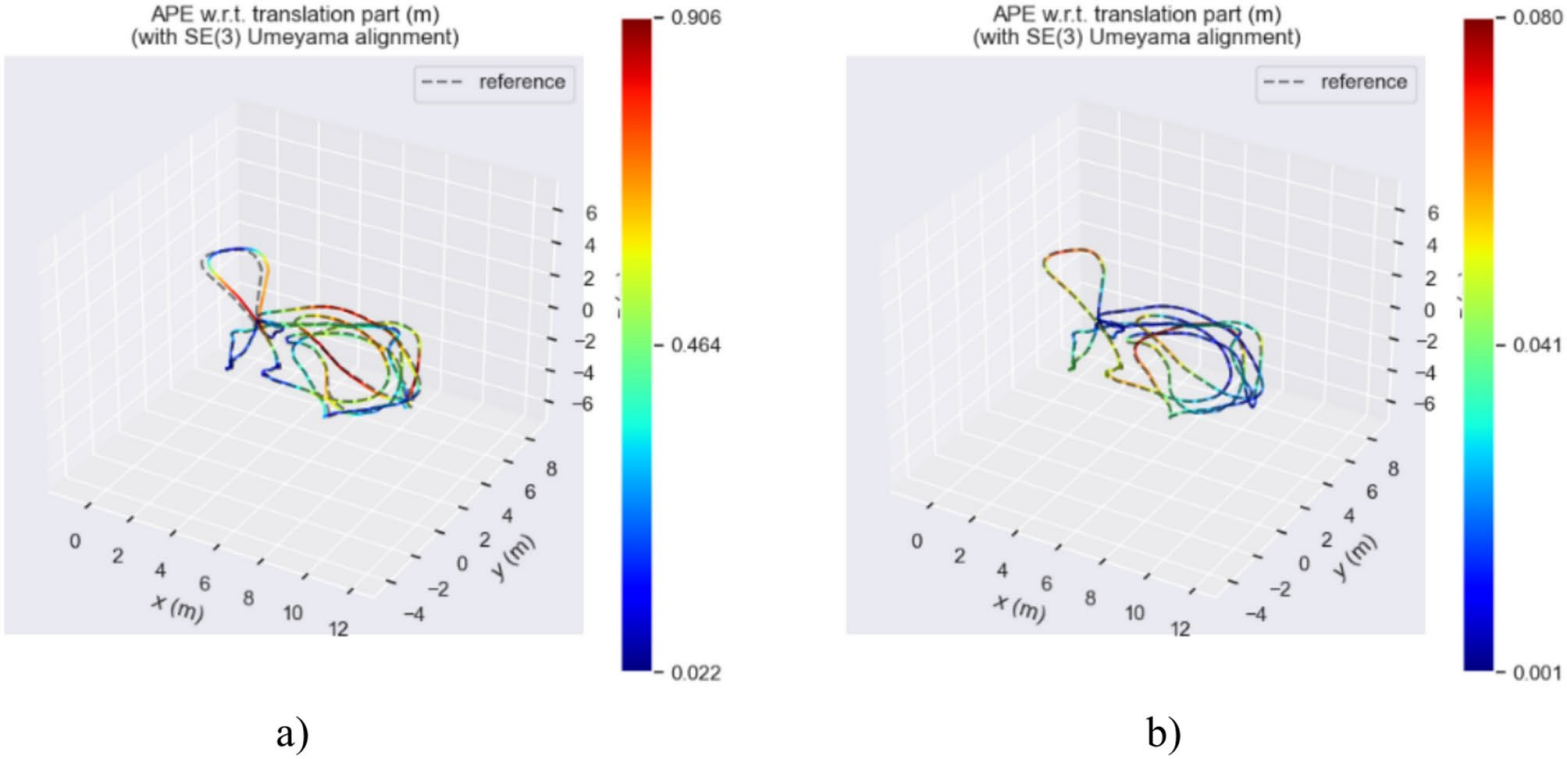
APE of trajectory estimation by two algorithms in MH_03_mediun sequence.

**Fig. 19:**
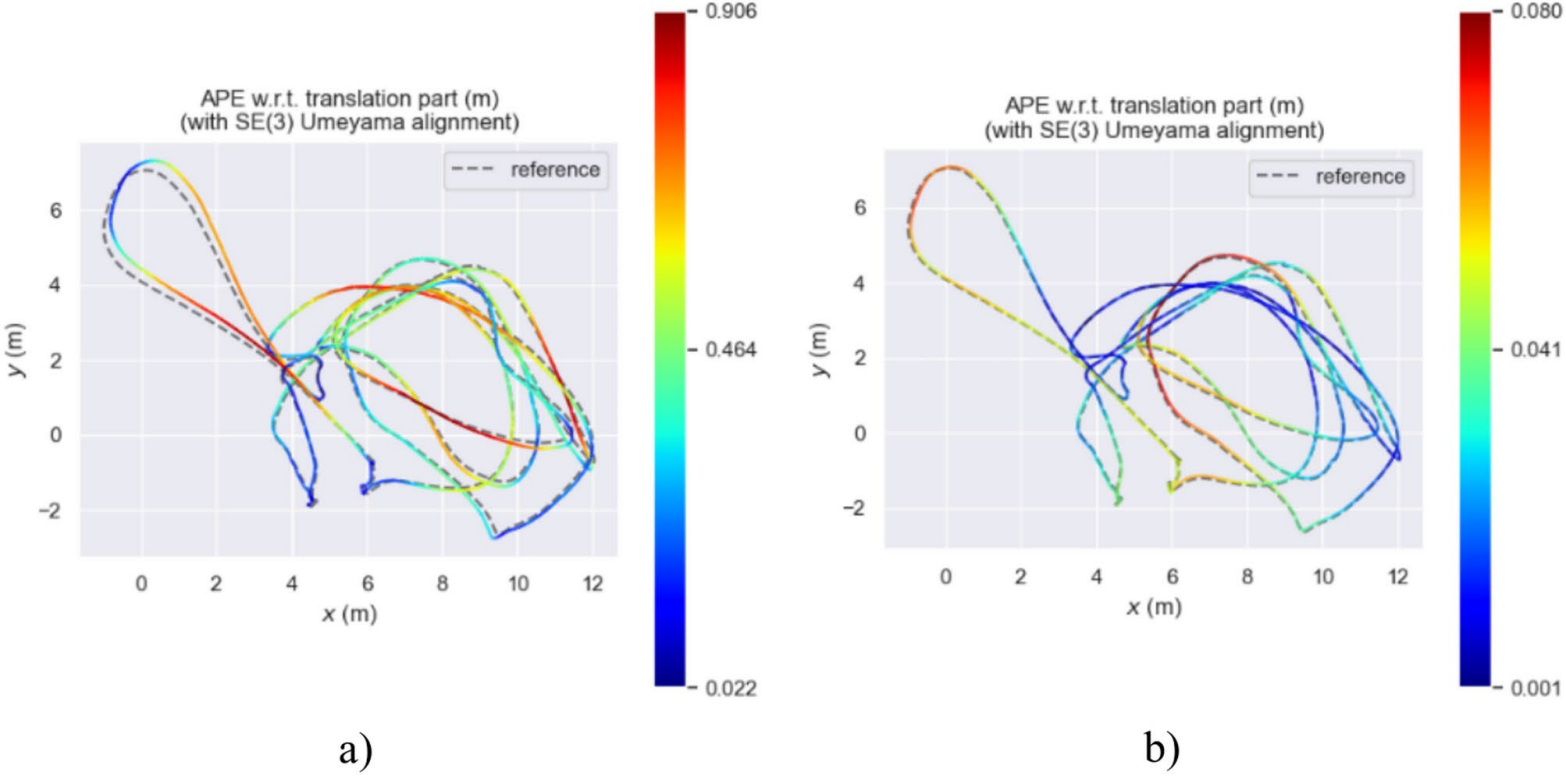
Two algorithms estimate the APE of the trajectory on the two-dimensional plane in the MH_03_mediun sequence.

**Fig. 20:**
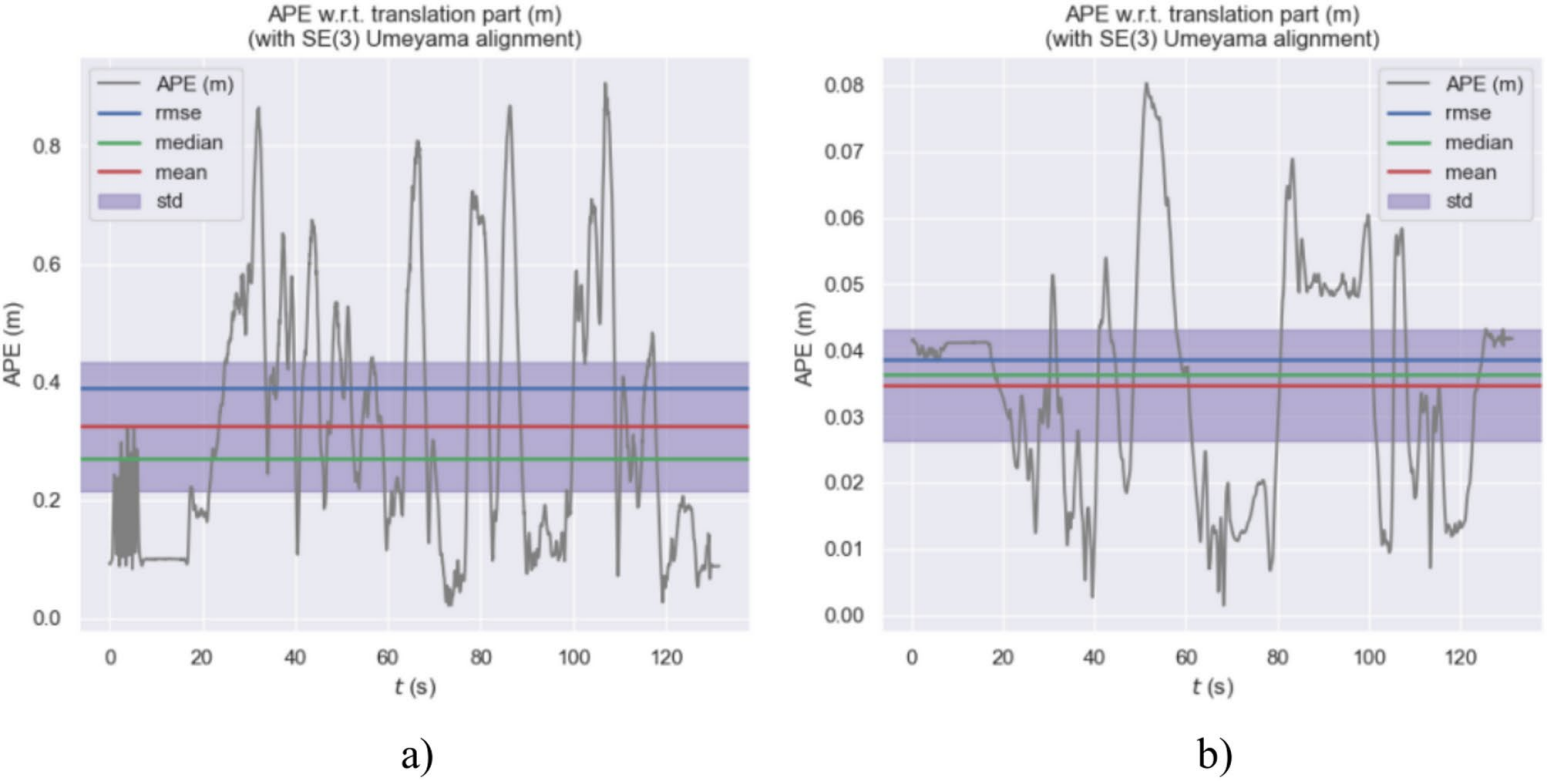
APE curve of estimated trajectories by two algorithms.

Figures 18, 19, and 20 display the APE (Absolute Pose Error) estimation results of the two algorithms for the trajectories. Observing Figs. 18 and 19, it’s clear that the algorithm proposed in this paper shows outstanding performance in trajectory estimation. The error in the estimated trajectory of our algorithm is considerably smaller than that of ORB-SLAM3, with only minor errors in very few cases. This fully demonstrates the superior effectiveness of our algorithm. Specifically, in Fig. 20, the APE curve shows an error gap of approximately 0.08 m and 0.85 m between our algorithm and ORB-SLAM3. This result once again confirms the superiority of our algorithm in trajectory estimation. Such performance advantages are significant for various applications requiring high-precision pose estimation. In summary, these experimental results demonstrate the excellent performance of our algorithm in trajectory estimation, having clear advantages in accuracy and stability compared to ORB-SLAM3.

Table 2 allows for the analysis of the performance of ORB-SLAM3 and our algorithm in three sequences. ORB-SLAM3’s maximum error range across all sequences is from 0.9060 to 2.2729 m, while that of our algorithm is from 0.0596 to 0.0803 m. ORB-SLAM3’s RMSE range across all sequences is from 0.2201 to 0.8278 m, while our algorithm’s RMSE range is from 0.0281 to 0.1575 m. Overall, according to the provided data, the performance of our algorithm across all sequences is significantly better than that of ORB-SLAM3.

**Table 2:** Euroc dataset ATE evaluation.

Sequence	Algorithms	Max/(m)	Min/(m)	RMES/(m)	Std/(m)
MH_01_easy	ORB-SLAM3	1.2323	0.0121	0.2201	0.1023
	Ours	0.0743	0.0023	0.0353	0.0190
MH_03_medium	ORB-SLAM3	0.9060	0.2188	0.3910	0.2177
	Ours	0.0803	0.0014	0.0385	0.0169
MH_05_difficult	ORB-SLAM3	2.2729	0.0363	0.8278	0.4487
	Ours	0.0596	0.0052	0.0309	0.0118

**Fig. 21:**
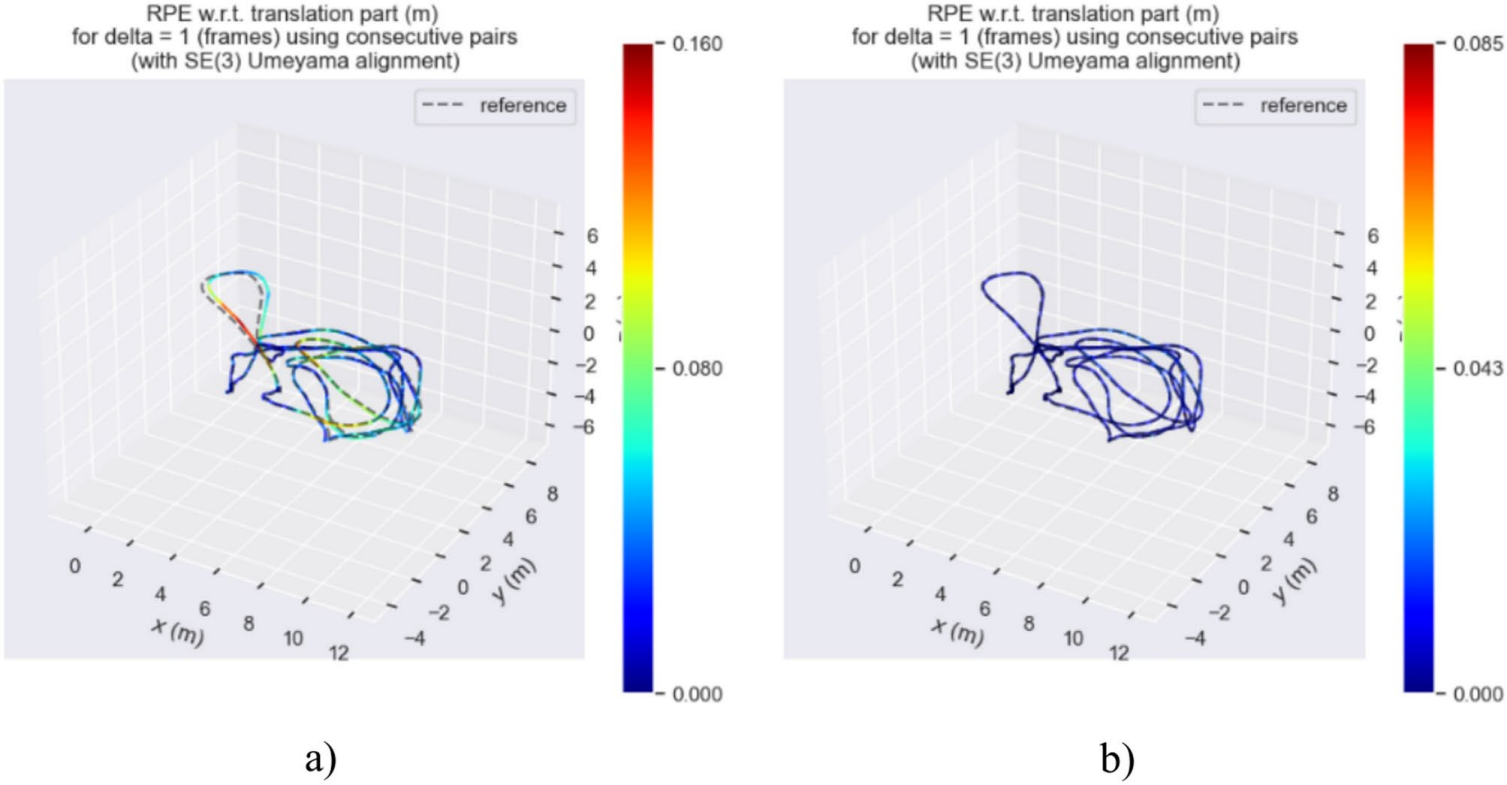
RPE of trajectory estimation by two algorithms in MH_03_mediun sequence.

**Fig. 22:**
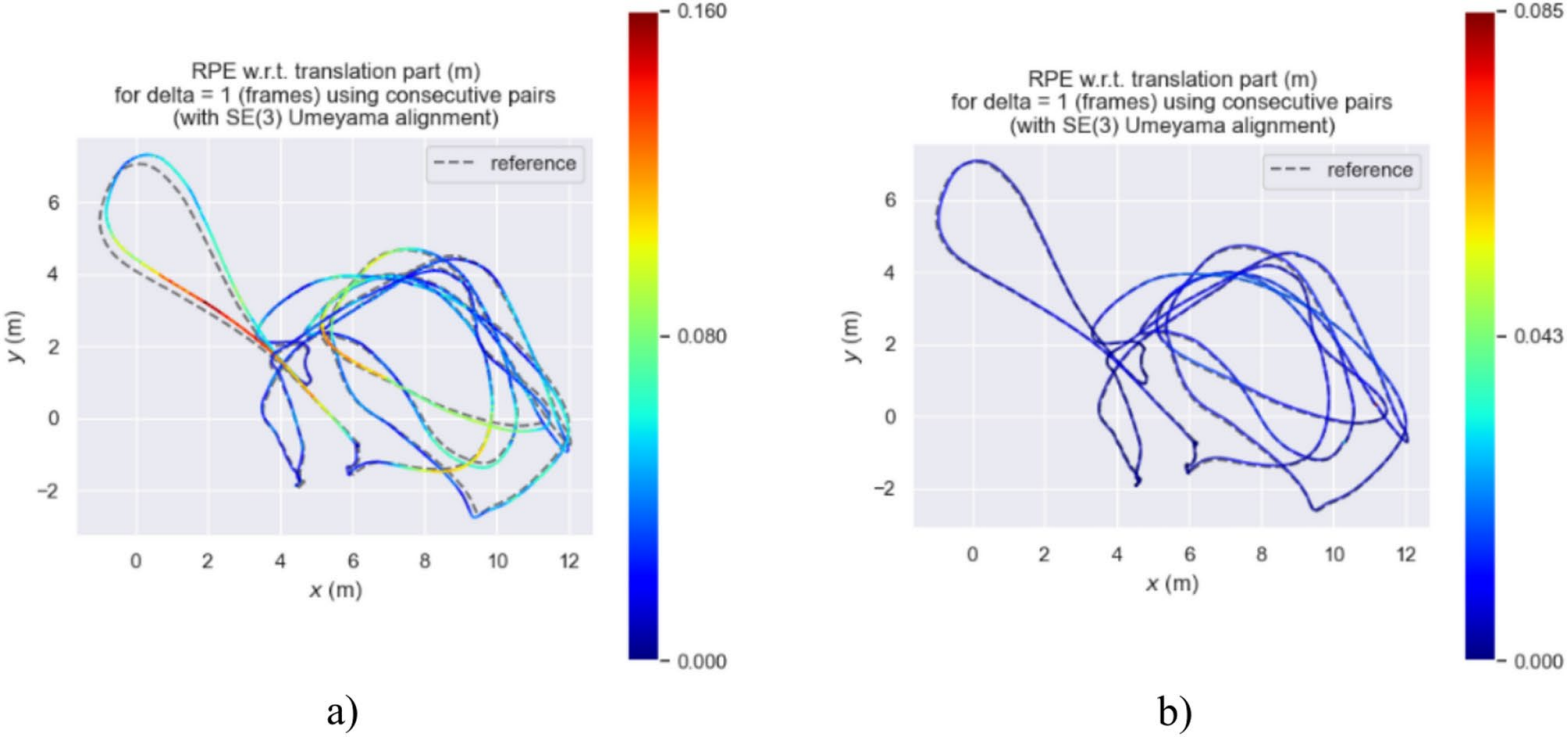
Two algorithms estimate the RPE of the trajectory on the two-dimensional plane in the MH_03_mediun sequence.

**Fig. 23:**
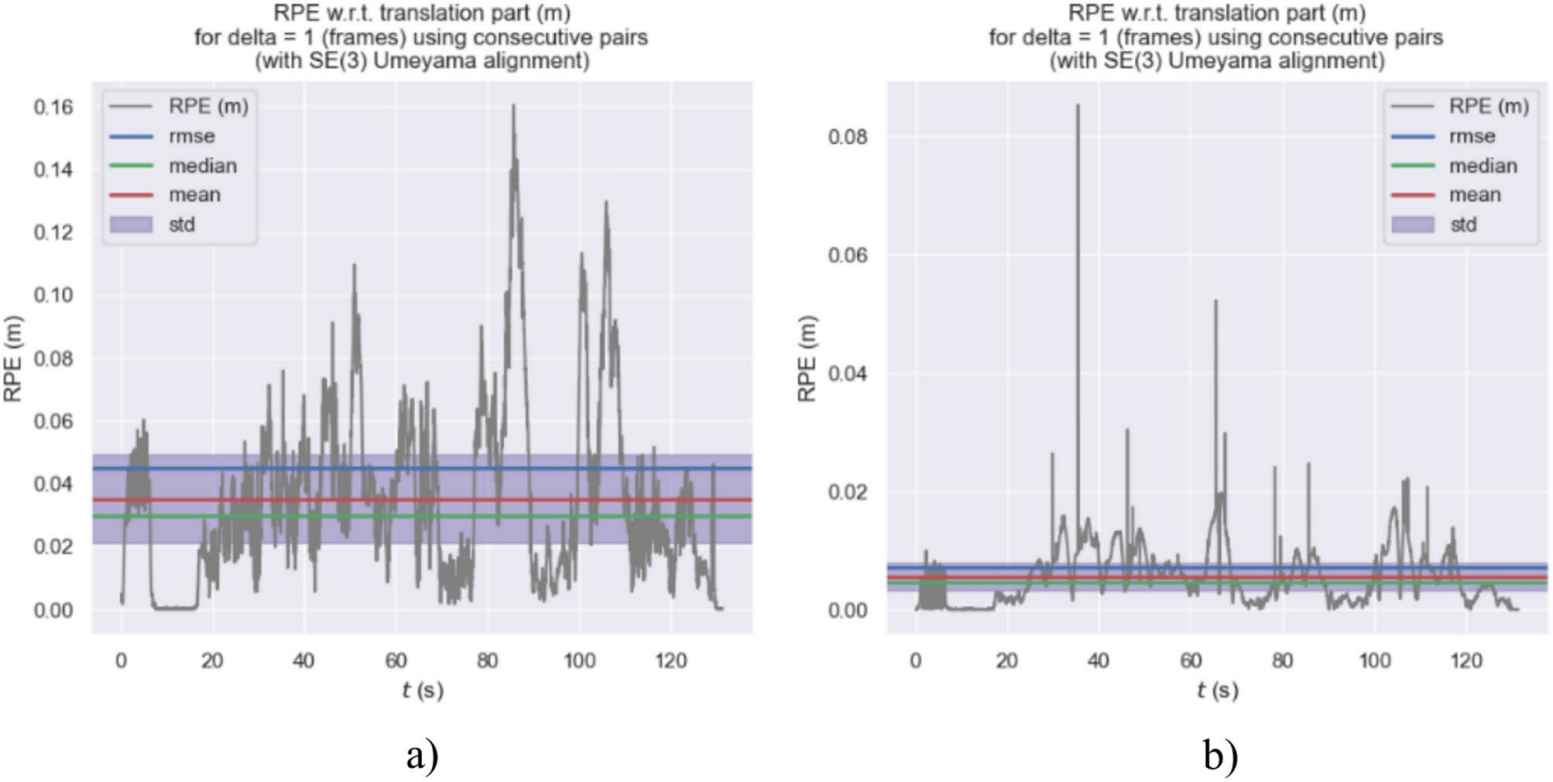
APE curve of estimated trajectories by two algorithms.

Figures 21, 22, and 23 show the RPE (Relative Pose Error) of the estimated trajectories of the two algorithms. From Figs. 21 and 22, it can be observed that the error of our algorithm is much smaller than that of ORB-SLAM3. In Fig. 23, the RPE curve indicates an error of approximately 0.16 and 0.085 m for our algorithm and ORB-SLAM3, respectively, validating the superiority of our algorithm.

Based on the RPE (Relative Pose Error) experimental data provided in Table 3, a detailed analysis of the performance of ORB-SLAM3 and our algorithm on three different data sequences (MH_01_easy, MH_03_medium, MH_05_difficult) is conducted. The maximum error range of ORB-SLAM3 on all these data sequences varies from 0.1604 to 1.3075 m, while the maximum error range of our algorithm on the same sequences is only between 0.0663 and 0.0978 m. Additionally, the RMSE (Root Mean Square Error) range of ORB-SLAM3 across all sequences varies from 0.0451 to 0.0803 m, whereas the RMSE range of our algorithm on the same sequences is from 0.0061 to 0.0281 m. These data indicate that our algorithm significantly outperforms ORB-SLAM3 on all data sequences, demonstrating improved resilience and enhanced location precision.

**Table 3:** Evaluation of RPE on the EuRoC dataset.

Sequence	Algorithms	Max/(m)	Min/(m)	RMES/(m)	Std/(m)
MH_01_easy	ORB-SLAM3	1.3075	0.000010	0.0554	0.0540
	Ours	0.0978	0.000008	0.0281	0.0163
MH_03_medium	ORB-SLAM3	0.1604	0.000025	0.0451	0.0282
	Ours	0.0852	0.000007	0.0071	0.0045
MH_05_difficult	ORB-SLAM3	0.2537	0.000008	0.0803	0.0499
	Ours	0.0663	0.000002	0.0061	0.0038

In summary, based on the analysis of the RPE experimental data, it can be concluded that our algorithm performs excellently on the three data sequences MH_01_easy, MH_03_medium, and MH_05_difficult. It has smaller maximum errors and lower RMSE, meaning that our algorithm can provide more accurate and stable relative pose estimation in processing these data sequences of varying difficulty levels. This performance advantage is of significant value for various application fields that require high-precision pose estimation. Although the proposed method performs well in multiple scenarios, it still has certain limitations. If dynamic objects cover more than 80% of the field of view (e.g., dense crowds), the lack of static features can lead to localization failure. Completely uniform surfaces such as plain white corridors make line feature extraction difficult; in custom monochrome environments, the number of line features drops to 3–5 per frame. In large-scale scenarios (e.g., the entire KITTI 00 sequence), the bag-of-words retrieval delay increases to 45 ms per frame. When running on a Jetson Nano, the frame rate drops to 12 FPS, requiring further optimization of binary descriptor computation.

To verify the adaptability of the proposed method in different scenarios, additional tests were conducted on the fr3/walking_xyz, fr3/sitting_static, and ICL-NUIM synthetic datasets. The TUM RGB-D dynamic scenes include disturbances such as pedestrian movement and object motion, while the ICL-NUIM synthetic dataset contains synthetic noise (Gaussian noise with σ = 15) and missing wall structures. In this context, DFMR represents the dynamic feature mismatch rate, and ATE denotes the absolute trajectory error.

According to Table 4, the proposed method demonstrates significant advantages across different scenarios. In the dynamic scene of TUM fr3/walking_xyz, the absolute trajectory error (ATE) of the proposed method is only 0.042 m, representing a 73.4% reduction compared to ORB-SLAM3 (0.158 m). The dynamic feature mismatch rate (DFMR) decreases from 34.7 to 12.3%, while the localization success rate improves to 91.7% (compared to the original 58.2%), indicating a substantial enhancement in dynamic interference suppression. In the ICL-NUIM noisy environment, the proposed method achieves an ATE of 0.098 m, a 51.7% reduction compared to PL-SLAM (0.203 m), with map completeness reaching 87.4% (up from 62.1%). These results verify the adaptability of line features in environments with missing structural information.

**Table 4:** Cross-dataset generalization capability comparison.

Dataset	Algorithms	ATE(m)	DFMR(%)	Success Rate
TUM fr3/walking_xyz	ORB-SLAM3	0.158	34.7	58.2
	Ours	0.042	12.3	91.7
ICL-NUIM Noise Scene	PL-SLAM	0.203		62.1
	Ours	0.098		87.4

Furthermore, to validate the independent effects of the adaptive AGAST algorithm and the point-line fusion-based loop closure detection, an ablation study was conducted, where the Baseline refers to the original ORB-SLAM3 framework.

As shown in Table 5, the comparison of different configurations on the EuRoC MH_05_difficult sequence highlights their impact on ATE and RPE: Baseline + AGAST reduces RMSE_ATE by 32.1% compared to the Baseline, validating the effectiveness of adaptive thresholding in optimizing low-texture environments. Baseline + LSD reduces RMSE_RPE by 21.4%, indicating that line features enhance structural constraints. The Full Model achieves an AP value 14.2% higher than Baseline + AGAST + LSD, proving that the Borda fusion strategy effectively suppresses single-modality mismatches.

**Table 5:** Comparison of ablation experimental performance.

Configuration	RMSE_ATE(m)	RMSE_RPE(m)	AP (%)
Baseline	0.8278	0.0803	68.2
Baseline + AGAST	0.5621	0.0645	73.8
Baseline + LSD	0.7012	0.0632	75.4
Baseline + AGAST + LSD	0.3987	0.0489	81.6
Full Model	0.0309	0.0061	95.3

## Conclusion

This paper adopts an adaptive AGAST point feature extraction algorithm combined with LSD line features to construct a point-line visual SLAM system. To further enhance the system’s robustness, a novel method for post-integration point-line incremental loop closure detection is proposed. This method, based on the Borda count strategy, fuses the similarity scores of point and line features. The proposed loop closure detection algorithm is compared with other loop closure detection methods, and its accuracy is verified using the classic KITTI dataset. A series of evaluation metrics are applied to comprehensively assess the system’s performance, with experimental comparisons conducted against the ORB-SLAM3 system on the EuRoC dataset. However, the algorithm still requires improvements in running speed. With current hardware capabilities, real-time processing of point-line SLAM systems in complex environments is not yet feasible. It is anticipated that with the ongoing development of computational hardware, future systems will process large-scale image data more efficiently.

The proposed point-line fusion SLAM algorithm demonstrates significant advantages in accuracy and robustness in low-texture environments, but there is still room for improvement. Future work can design a parallel optimization framework based on CPU-GPU heterogeneous computing, such as offloading line feature matching and descriptor computation to the GPU (e.g., CUDA acceleration) while using CPU multi-threading for feature extraction and loop closure detection. For embedded devices (e.g., drones, mobile robots), the system can dynamically adjust the number of point-line feature extractions based on scene complexity (e.g., prioritizing line features in low-texture environments) or compress BEBLID and LBD descriptors from 128-bit to 64-bit for fast matching using Hamming distance. In terms of hardware, integrating FPGA or ASIC chip designs can achieve hardware-level optimization for key modules such as adaptive AGAST threshold calculation and LSD line detection, improving energy efficiency.

To enhance robustness against dynamic objects, a lightweight semantic segmentation model (e.g., MobileNetV3) can be integrated to distinguish static backgrounds from dynamic objects (such as pedestrians and vehicles), ensuring that feature matching and optimization are only performed on static regions. Additionally, IMU or wheel odometry can provide motion priors, which, when combined with RANSAC, help eliminate dynamic feature mismatches.

In autonomous driving, the CTAFFNet^50^ method proposes a novel CNN-transformer adaptive feature fusion network for object detection and develops an adaptive feature fusion strategy that combines local high-frequency and global low-frequency features to obtain comprehensive feature information. This approach can be integrated with our method to achieve better results. In agricultural and industrial automation, service robots can leverage the fusion of line and point features to address challenges in map maintenance under frequent dynamic object interference. The scene structure information constructed by SLAM can assist robots in understanding the spatial semantics of human gestures or voice commands.

## Data Availability

The datasets analysed during the current study are available in the KITTI and EuRoC repository, 10.1177/0,278,364,913,491,297, 10.1177/0,278,364,915,620,033.
